# Transition paths across the epithelial-mesenchymal transition landscape are dictated by network logic

**DOI:** 10.1242/dev.204583

**Published:** 2025-06-12

**Authors:** Anupam Dey, Adam L. MacLean

**Affiliations:** Department of Quantitative and Computational Biology, University of Southern California, Los Angeles, CA 90089, USA

**Keywords:** Gene regulatory network, Multistability, Tristability, EMT, Network logic, Mathematical modeling

## Abstract

During development and oncogenesis, cells transition reversibly from epithelial to mesenchymal states (epithelial-mesenchymal transition; EMT). Tristable EMT can be described by a three-node gene regulatory network in which multiple inputs regulate the transcription factor ZEB. In this network, and more generally, it is not known how to choose combinatorial network logic. Here, we discover that the choice of multiplicative (AND) versus additive (OR) network logic strongly affects EMT phenotypes and leads to opposing predictions regarding factors that control EMT transition paths. We show that strong inhibition of miR-200 destabilizes the epithelial state and initiates EMT for AND logic, in agreement with experimental data. Using single-cell data, stochastic simulations and perturbation analysis, we show how these results can be used to design experiments to infer EMT network logic in live cells. We go on to analyze networks controlling cell fate decisions during embryogenesis and show that, here too, logic changes cell fate landscapes upon perturbation in important ways. Our results stress the importance of considering logic in the construction of models of regulatory networks that govern cell fate decisions.

## Teaser

**Summary:** Mathematical models of gene regulatory networks make assumptions about how signals combine logically; great caution must be taken in this choice of logic, as it can lead to divergent phenotypes.

## INTRODUCTION

The fate of a cell is determined by its transcriptional state, which is in turn determined by a gene regulatory network (GRN). GRNs consist of signed (activating or inhibitory) directed interactions between transcription factors, genes and other genetic elements (Britten and Davidson, 1969). Constructing ‘complete’ GRNs that describe biological function is typically beyond our grasp. With a few possible exceptions, such as sea urchin development (Davidson et al., 2002) or partial mammalian networks (e.g. in hematopoiesis; Schütte et al., 2016), constructing whole-organ GRNs remains a grand challenge. Moreover, GRNs quickly grow large and contain many redundancies (Iwasa, 2023). Rather than seeking to construct full networks, small GRN (i.e. 2-5 nodes) network ‘motifs’ (Ma et al., 2009; Alon, 2020) can explain expected phenotypes with low-dimensional dynamics (Tripathi et al., 2023). Geometrical models offer the means to explain low-dimensional dynamics by providing a bridge between the metaphorically smooth Waddington landscape (Waddington, 1957) and quantitative measurements of single-cell states (Rand et al., 2021; Sáez et al., 2022). A common challenge in the construction of models described by small network motifs is the choice of network logic.

Epithelial-mesenchymal transition (EMT) is a quintessential cell state transition, and can be characterized by small GRNs (Hong and Xing, 2024). Various GRN models of EMT have been proposed, most of which share a core network comprising the transcription factors ZEB and SNAIL, and the micro-RNAs miR-200 and miR-34 (Hong et al., 2015; Tian et al., 2013; Zhang et al., 2014; Lu et al., 2013; Jolly et al., 2016). The core network consists of mutual inhibitory feedback loops between ZEB-miR-200 and SNAIL-miR-34. Expression of miR-200 maintains the epithelial state, while ZEB (representing the transcription factors ZEB1 and ZEB2) induces EMT. ZEB can be activated by SNAIL, which itself is inhibited by miR-34. Models have shed light on the dynamics and the cell states accessible during EMT: in particular, at least three stable steady states can exist during EMT and cells often transition through one or more EMT intermediate cell states. EMT intermediate states are also referred to as partial EMT states or hybrid E/M states (Sha et al., 2019). In addition, EMT can proceed through multiple different transition paths (Wang et al., 2022).

To construct a mathematical model from a GRN (described by ordinary or stochastic differential equations), a choice of network logic must be made when a gene receives more than one regulatory input. The model of Tian et al. (2013), for example, is constructed with additive gene regulation (OR logic), whereas the model of Lu et al. (2013) is multiplicative (AND logic). There is, in general, no principled guidance on the choice of network logic when constructing a mathematical model. Indeed, recent work has shown that signals combine to regulate gene expression in both additive and multiplicative manners at roughly equal proportions (a variety of other responses are also observed at lower frequency) (Sanford et al., 2020). The gene-level specificity of the responses measured by Sanford et al. (2020) (for two specific input signals: retinoic acid and TGFβ) emphasizes the challenge in choosing network logic to model a GRN. Furthermore, the impact of this choice on gene regulatory dynamics and the resulting cell fate landscape is for the most part unknown.

In this work, we investigate how the choice of network logic impacts gene regulatory dynamics and cell state transitions during EMT. We do so via a GRN that permits tristable EMT, consisting of SNAIL, miR-200 and ZEB (Lu et al., 2013; Jia et al., 2017). We discover that EMT phenotypes are sensitive to the choice of logic. In the next section, we introduce the GRN model and discuss the transition paths it permits. We go on to show that the EMT regulatory parameters have opposing effects on the landscape, depending on the choice of logic. We first demonstrate this for a constrained model with comparable interaction strengths, and then show that our results hold for a wide range of models with unconstrained parameters. We discuss our results on logic in light of the combinatorics of gene regulation and show how EMT GRN logic can be inferred from experimental data. Finally, we discuss the role of logic in cell fate decisions beyond EMT. For GRNs controlling developmental cell fate decisions, we show – in agreement with results for the miR-200-ZEB regulatory network – that while different logics can produce similar bifurcation landscapes, they can produce dramatically different dynamics on these landscapes: logic must be carefully considered during model construction.

## RESULTS

### Analyzing logic choice in a three-node regulatory network of EMT

**Fig. 1.**
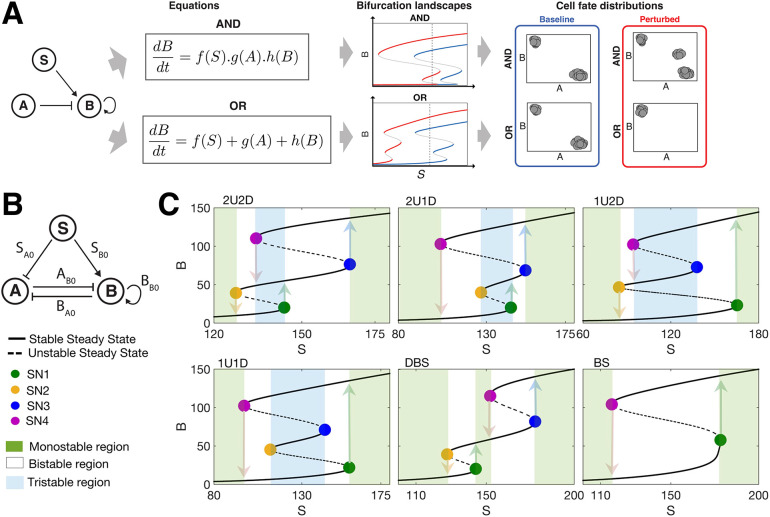
**Multistable responses generated by a three-node EMT network.** (A) Schematic overview of the methodological approach: a choice must be made when constructing ODE models from GRNs as to whether edges combine multiplicatively (AND) or additively (OR). Based on this choice, one can analyze the bifurcation landscapes and cell fate distributions that result. Even when baseline landscapes generated by each logic (blue) look similar, perturbations (red) can induce very different landscapes and cell fate distributions. (B) The three-node EMT network described by input signal SNAIL (*S*), miR-200 (*A*) and ZEB (*B*). Parameters (*S*_*A*0_, *S*_*B*0_, *A*_*B*0_, *B*_*A*0_, *B*_*B*0_) denote the half-maximal concentrations of species that (positively or negatively) regulate another species in the network. (C) All possible multistable responses in *B* with respect to *S*. Four types of tristable response and two bistable responses exist; colored circles indicate saddle node (SN) points and arrows indicate transition paths. The number of upward transitions marked as 1U or 2U; similar for downward transitions (1D or 2D). DBS, double bistable switch; BS, bistable switch.

To construct an ODE model for a GRN, one must choose how multiple inputs regulating a gene affect its expression. With respect to multiple inputs, AND logic describes responses that are multiplicative, and OR logic describes responses that are additive. We will analyze the impact of this choice in terms of the cell state transition dynamics (trajectories) and the multistability landscapes (bifurcations) that arise (Fig. 1A).

To study the impact of logic on EMT, we investigated a three-node GRN that was originally developed by Lu et al. (2013). The GRN consists of SNAIL (*S*), which acts as an input signal, miR-200 (*A*) and ZEB (*B*). *A* and *B* mutually inhibit each other. *S* inhibits *A* and activates *B*; and *B* also has a self-activation (Fig. 1B). Of note: in this network, *B* can be both directly activated by *S* and indirectly activated by *S* via a double inhibition: 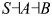. The direct and indirect activation pathways form a coherent feed-forward loop (Alon, 2020) that regulates *B* in combination with two additional loops: mutual inhibition between *A* and *B*, and self-activation of *B*. Modeled by ordinary differential equations (ODEs) with Hill function kinetics, this model permits bistability or tristability under certain conditions.

In constructing an ODE model for this network, one must choose whether gene regulations combine additively or multiplicatively (Fig. 1A). This choice can be approached either phenomenologically or mechanistically, where, in the former case, one can use experimental observations to infer logic (e.g. as in Bessonnard et al., 2014); in the latter case (constructing models from first principles via mechanisms of transcription), models that appeal to thermodynamic principles to describe the action of RNA polymerase lead to multiplicative regulation (Bintu et al., 2005) and models that alternatively assume transcription factors more commonly bind independently to regulate transcription lead to additive regulation (Sanford et al., 2020).

There is little principled guidance in the literature regarding how to make this choice practically. For example, comparison of two well-studied models of EMT shows that Lu et al. (2013) model the GRN with AND logic, whereas Tian et al. (2013) model the GRN with OR logic. Moreover, appealing to experimental evidence does little to help: a deep investigation into the effects of two regulatory signals combined showed that the downstream effects of their synergy on target gene expression led to both additive and multiplicative responses, observed in almost equal proportions (as well as, less commonly, sub-additive or super-multiplicative responses) in an interaction-specific manner (Sanford et al., 2020). Thus, it is difficult to make principled guesses *a priori*, and to be confident in any particular choice would require measurement of specific gene-gene interactions in response to a specific stimulus. To account for this uncertainty, we consider ODE models of the same GRN constructed with alternative logic, with the AND model given by:
(1rma)

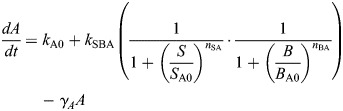

(1rmb)

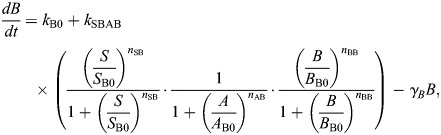
and the OR model given by:
(2rma)

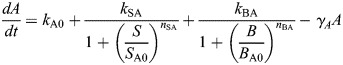

(2rmb)

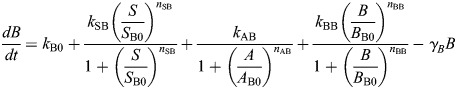
where the parameter descriptions and values used are given in Table 1, and discussed further in the Materials and Methods.

**Table 1.** Description of model parameters and values

Notation	Parameter description	Constrained models	Unconstrained models	Range
*k*_A0_, *k*_B0_	Basal synthesis rate	*k*_A0_=*k*_B0_	*k*_A0_≠*k*_B0_	[0.1,1]
*k*_SBA_, *k*_SBAB_	Maximal expression of *A* via regulation from *S* & *B*, maximal expression of *B* via regulation from *S*, *B* & *A* (AND model)	*k*_SBA_=*k*_SBAB_	*k*_SBA_≠*k*_SBAB_	[1,100]
*k*_SA_, *k*_SB_	Maximal expression of *A*, *B* via regulation from *S* (OR model)	*k*_SA_=*k*_SB_	*k*_SA_≠*k*_SB_	[1,100]
*k*_BA_, *k*_AB_	Maximal expression of *A* via regulation from *B*, and vice versa (OR model)	*k*_BA_=*k*_AB_	*k*_BA_≠*k*_AB_	[1,100]
*S*_A0_, *S*_B0_	Strength of regulation from *S* to *A*, *B*	*S*_A0_=*S*_B0_	*S*_A0_≠*S*_B0_	[10,500]
*B*_A0_, *A*_B0_	Strength of inhibition from *A* to *B*, and vice versa	*B*_A0_=*A*_B0_	*B*_A0_≠*A*_B0_	[10,500]
*n*_SA_, *n*_SB_	Hill coefficient for *A*, *B* due to regulation from *S*	*n*_SA_=*n*_SB_	*n*_SA_≠*n*_SB_	[1,10]
*n*_AB_, *n*_BA_	Hill coefficient for *A* due to inhibition from *B*, and vice versa	*n*_AB_=*n*_BA_	*n*_AB_≠*n*_BA_	[1,10]
*γ*_A_, *γ*_B_	Degradation rate of *A*, *B*	*γ*_A_=*γ*_B_	*γ*_A_≠*γ*_B_	[0.01,0.1]
*k*_BB_	Maximal expression of *B* via self-regulation	–	–	[1,100]
*B*_B0_	Strength of self-regulation of *B*	–	–	[10,500]
*n*_BB_	Hill coefficient for self-regulation of *B*	–	–	[1,10]

The first column gives the parameter notation, the second column is the parameter description, the third and fourth columns give relationships between parameters under different models, and the fifth column gives the parameter range used for sampling. Hill coefficients take integer values; all other parameters take real values.

Each of these models of the EMT GRN (Fig. 1B), defined either by AND (Eqn 1) or OR (Eqn 2) logic, permits tristability. The EMT GRN is similar to a minimal network that permits tristability, consisting of two coupled positive-feedback loops with ultrasensitivity, which is found in EMT and other biological contexts (Dey and Barik, 2021; Frankhouser et al., 2024). For each tristable model, six multistable responses to changes in the input signal *S* can be defined: four tristable and two bistable (Fig. 1C). The saddle-node (SN) points then define the transition points between states. Elsewhere in development, similar multistable dynamics can be characterized to represent, for example stem/progenitor cell differentiation.

Of the SN points: SN1 marks the EMT initiation point and SN4 marks the mesenchymal-epithelial transition (MET) initiation point. Denoting upward transitions (with increasing *S*) as ‘U’ and downward transitions (with decreasing *S*) as ‘D’, there can be one or two upward transitions (1U or 2U) and likewise for downward transitions (1D or 2D). Thus, for the overall landscape (considering EMT and the reverse MET together), there are four tristable paths: the 2U2D path passes through the hybrid E/M state during both EMT and MET, whereas the 1U1D path does not pass through the E/M state in either direction. The 2U1D path passes through E/M state during EMT but not during MET, and vice versa for the 1U2D path. Of the two bistable EMT landscapes: one is a simple bistable switch (BS) and the other is a double bistable switch (DBS). The double bistable switch has a hybrid E/M state that is monostable between two regions of bistability: of all EMT landscapes, this has the most accessible/stable hybrid state, since here for some range of *S* only the hybrid state exists.

For the EMT network modeled by either AND (Eqn 1) or OR logic (Eqn 2), we have seen that different tristable landscapes exist, i.e. each model permits the co-existence of epithelial, mesenchymal and hybrid cell states with different possible transition paths between them. Below, we go on to test whether, given different choices of logic, different conclusions will be drawn.

### Logic controls the effects of direct vs indirect ZEB activation on EMT

**Fig. 2.**
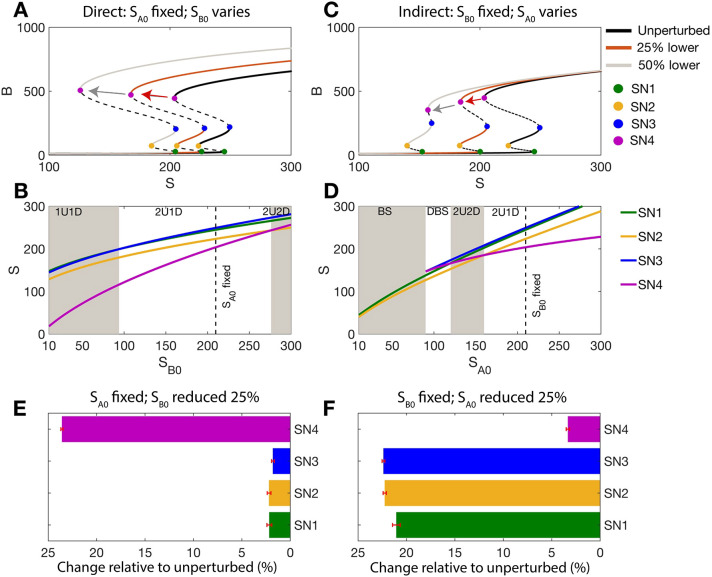
**Impacts on EMT of varying regulation strength for AND logic models.** (A) Impact on tristable EMT landscapes for the AND model when varying the direct activation strength (*S*_B0_): unperturbed network and networks perturbed by 25% (red arrow and curve) or 50% (gray arrow and curve). (B) The loci of the SN points as a function of *S*_B0_. (C) As for panel A, when varying the indirect activation strength (*S*_A0_): unperturbed network and networks perturbed by 25% or 50%. (D) The loci of the SN points as a function of *S*_A0_. (E) Sensitivity of the SN points for 150 EMT networks perturbed by a 25% increase in the direct activation strength (*S*_B0_ reduced by 25%). (F) Sensitivity of the SN points for 150 EMT networks perturbed by a 25% increase in the indirect activation strength (*S*_A0_ reduced by 25%).

**Fig. 3.**
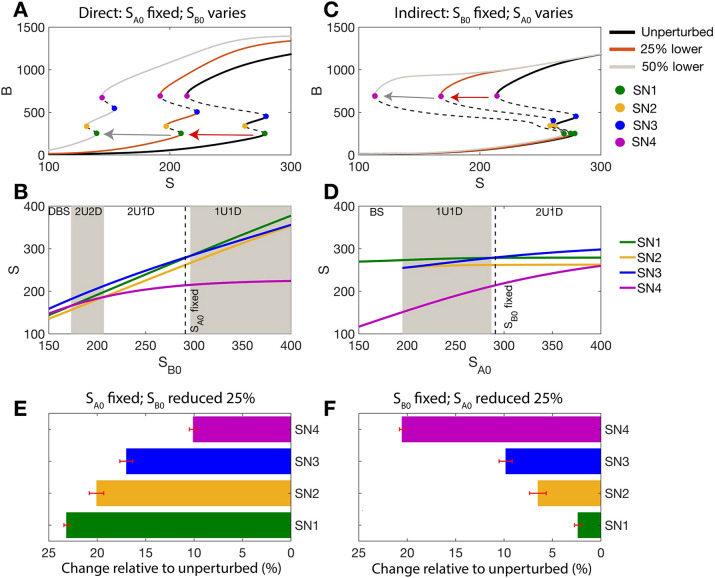
**Impacts on EMT of varying regulation strength for OR logic models.** (A) Impact on tristable EMT landscapes for the OR model when varying the direct activation strength (*S*_B0_): unperturbed network and networks perturbed by 25% (red arrow and curve) or 50% (gray arrow and curve). (B) The loci of the SN points as a function of *S*_B0_. (C) As for panel A, when varying the indirect activation strength (*S*_A0_): unperturbed network and networks perturbed by 25% or 50%. (D) The loci of the SN points as a function of *S*_A0_. (E) Sensitivity of the SN points for 108 EMT networks perturbed by a 25% increase in the direct activation strength (*S*_B0_ reduced by 25%). (F) Sensitivity of the SN points for 108 EMT networks perturbed by a 25% increase in the indirect activation strength (*S*_A0_ reduced by 25%).

To reveal how network logic affects transition paths during EMT and MET, we studied EMT landscapes under perturbations of the direct activation of ZEB (*B*) and the indirect activation of ZEB via inhibition of miR-200 (*A*) for an AND versus OR network logic. We perturbed constrained models with symmetrical regulations (i.e. *S* regulates *A* and *B* equally; *A* and *B* inhibit each other equally, etc.; for full details, see Materials and Methods). The direct activation strength (*S*_*B*0_) and the indirect activation strength (*S*_*A*0_) were each perturbed by x% from *S*_*A*0_=*S*_*B*0_. The resulting EMT landscapes were analyzed for AND (Fig. 2) and OR models (Fig. 3).

For AND models, we increased the direct (Fig. 2A) or the indirect (Fig. 2C) activation of *B* by 25% from parity (*S*_*A*0_=*S*_*B*0_; see Materials and Methods). Note that, due to the form of the equations, we refer to a decrease in the parameter value by x% as an increase in the strength of regulation via that parameter by x%. Increasing the direct activation rate shifted the whole bifurcation to the left with SN4 (pink) shifting the most compared to SN1-SN3 (Fig. 2A,B). Fig. 2B depicts the loci of the four SN points as a function of the direct activation strength (*S*_*B*0_). SN4 is most sensitive to changes in the direct activation strength. Increasing the direct activation strength also changes the tristable response type from 2U1D to 1U1D, indicating that the hybrid (E/M) state loses stability as the direct activation strength is increased.

When the indirect activation strength *S*_*A*0_ was increased by the same amount, the bifurcation once again shifted to the left, but in this case SN4 shifted the least. SN1 shifted the farthest and is thus the most sensitive to the indirect activation strength (Fig. 2C,D). Fig. 2D also shows that, as the indirect strength increases (lower *S*_*A*0_), the EMT landscape loses tristability and forms a bistable switch. Overall, we see that SN points change according to the perturbation: increasing the indirect activation parameter (Fig. 2C) leads to a much larger change in SN1 than increasing the direct activation parameter by the same amount (Fig. 2A). Thus, EMT is initiated early (with lower *S*) as the indirect regulation strength is increased, as compared to an equivalent increase in the direct regulation strength.

The results so far apply to only one set of parameter values. To test their generality, we generated many tristable AND models. To do so, we used a method for analysis of model multistability previously described (Dey and Barik, 2021) (see Materials and Methods). For each tristable parameter set, we varied the direct or indirect activation strength by 25% and retained only those parameter sets that generated tristability during both these perturbations. Out of 300 total parameter sets for AND models, 150 permitted tristability under both perturbations. For all 150 tristable parameter sets, we observed that varying the direct activation rate led to large changes in SN4 relative to the other SN points (Fig. 2E), whereas varying the indirect activation rate led to large changes in SN1-SN3 relative to SN4 (Fig. 2F). For the AND model, SN4 is thus most sensitive to changes in the direct activation rate and least sensitive to changes in the indirect activation rate. SN1, on the other hand, is most sensitive to changes in the indirect activation rate, suggesting that the indirect activation of ZEB more strongly regulates the initiation of EMT.

An equivalent analysis of direct and indirect activation parameters was performed for the model constructed with OR logic; the results showed opposing effects to those of the AND model. For the OR model, when the direct activation rate was increased by 25% (*S*_*B*0_ lowered), SN1 was the most sensitive and SN4 was the least sensitive point (Fig. 3A,B). When the indirect activation strength was increased by 25% (*S*_*A*0_ lowered), SN4 was the most sensitive, in contrast to the observation for AND models (Fig. 3C,D). Analysis of all parameter sets permitting tristability after perturbing OR models (108/300 parameter sets) showed that SN1 is most sensitive to the direct activation strength (Fig. 3E) and that SN4 is most sensitive to the indirect activation strength (Fig. 3F). Thus, for OR models, the direct activation parameter initiates EMT, as SN1 is most sensitive to this parameter.

We have shown that different choices of logic lead to differences in the impact of regulations on ZEB in the EMT network. Epithelial state stability and the initiation point of EMT are sensitive to changes in miR-200 inhibition for AND logic, but to changes in the ZEB activation rate for OR logic. Not only is the initiation point of EMT sensitive to different regulatory parameters depending on the choice of logic, but different choices of logic can lead one to opposing conclusions about the transition paths of cells undergoing EMT.

### The role of network logic is conserved for a wide family of models

**Fig. 4.**
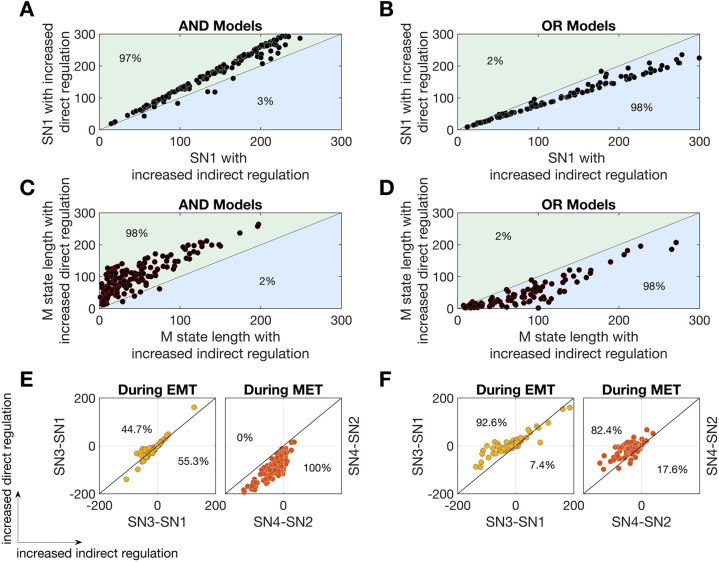
**Divergent features of tristable EMT landscapes for AND versus OR models.** (A,B) Scatter plots of SN1, which is the EMT initiation point, for perturbations to AND models (A) and OR models (B). Constrained tristable models for both logics were perturbed by 25% along each axis (direct or indirect regulation). (C,D) Scatter plots of the M state length for perturbations to AND models (C) and OR models (D). Solid line drawn for *y*=*x*. (E,F) Scatter plots showing the accessibility to the hybrid state during EMT and MET during 25% direct and indirect perturbations for the AND models (E) and OR models (F). The distance between SN3 and SN1 is a measure of the accessibility of the hybrid state during EMT; the distance between SN4 and SN2 is a measure of the accessibility of the hybrid state during MET. Larger values indicate increased accessibility.

We next analyzed properties of the EMT landscape over a wide range of parameter values that permit tristability. Analysis of EMT phenotypes via three landscape features: the EMT initiation point (saddle node point SN1; Fig. 1C), the length of the mesenchymal state and the accessibility of the hybrid state (see supplementary Materials and Methods, section 1), showed that different logic choices led to divergent phenotypes (Fig. 4). For AND models, increasing miR-200 inhibition destabilized the epithelial and mesenchymal states, and increased the accessibility of the hybrid state. For OR models, increasing miR-200 inhibition stabilized the epithelial and mesenchymal states, and delayed the initiation of EMT. A considerable body of literature on EMT and miR-200 suggests that suppressing miR-200 is sufficient to initiate EMT, and that re-expressing miR-200 in mesenchymal cells can initiate MET (Korpal et al., 2008; Nagai et al., 2024; Kong et al., 2009; Zhang et al., 2012). In light of these experimental studies and our results on the alternative phenotypes observed with different network logic, the EMT GRN appears most consistent with AND logic.

Through a widespread analysis of larger perturbations (Fig. S1), alternative parameters of the network (Fig. S2), models with unconstrained parameter values (Fig. S3) and alternative formulations of the model (Fig. S4), we found strong evidence for the generality of our results (see supplementary Materials and Methods, section 2). We investigated other parameters: the inhibition of *B* from *A* acted similarly to the inhibition of *A* from *S*. However, neither the self-activation of *B* nor the inhibition on *A* from *B* were affected by logic (Figs S5 and S6). Furthermore, we analyzed how multiple inputs combine differentially to produce these results (Fig. S7; see supplementary Materials and Methods, section 3) and studied the impact of an expanded network incorporating mRNA species into the model (Fig. S8). Overall, these results reveal that, across many different parameterizations of the core EMT network, and even for alternative model constructions, the effects of the direct and/or indirect ZEB activation parameters (but not other network parameters) are dictated by the logic with which the model is constructed.

### Experimental design can reveal the network logic in cells undergoing EMT

The opposing effects of AND versus OR logic on EMT phenotypes can be analyzed in light of data to inform experimental design and reveal how EMT transcriptional networks are wired in specific cell types. Zhang et al. (2022) used a transformed human mammary epithelial cell line (HMLER) to create single-cell clones that displayed markedly varied EMT phenotypes when maintained in culture. Approximately 3/4 of the single-cell-derived clones were non-convertible: they stably maintained their epithelial status in culture. In contrast, 1/4 of the clones were convertible: they were able to spontaneously undergo EMT and displayed a spectrum of EMT states, including hybrid E/M cells.

**Fig. 5.**
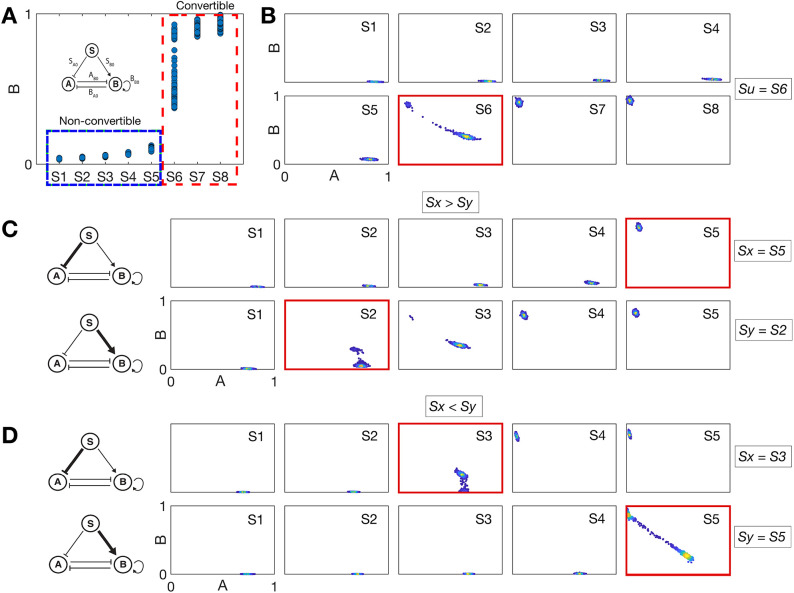
**Experimental design reveals the network logic of GRNs for EMT.** (A) Stochastic simulations of the EMT GRN with multiplicative noise for different *S* values can categorize cells as non-convertible (signal levels *S*≤*S*_5_, blue outline) or convertible (signal levels *S*≥*S*_6_, red outline). (B) Equilibria of the model in the (*A*, *B*) phase plane for stochastic differential equation simulations of the unperturbed model. *S*_*i*_ (where *i* ∈ [*x*, *y*, 1, 2, 3, …, 8]) indicates an input signal strength of signal *S*; red outline indicates lowest signal strength at which cells become convertible. (C) Equilibria of the model in the (*A*, *B*) phase plane for simulations of the OR logic GRN. Either the indirect (top row) or the direct (bottom row) regulation on ZEB is perturbed by 25% and the minimum signal required to initiate EMT is recorded (*S*_*x*_ for indirect and *S*_*y*_ for direct). (D) Equilibria of the model in the (*A*, *B*) phase plane for simulations of the AND logic GRN, perturbing either the indirect or the direct regulation, as for panel C.

To compare model-predicted EMT phenotypes with the tristable responses observed in live HMLER cells (Zhang et al., 2022), we studied stochastic EMT transition paths for the miR200-ZEB network simulated via stochastic differential equation (SDE) models (see Materials and Methods). We simulated SDEs specified by either multiplicative or additive noise (see Materials and Methods) to analyze the effects of each on the cell state landscape (Fig. 5 and Fig. S9). Whereas additive noise is simpler to quantify and study, multiplicative noise may occur more often in biological systems. Caution must be taken, however, since it has been shown that multiplicative noise can also alter the cell fate landscape (Coomer et al., 2021).

For low levels of signal *S*, cells cannot transition out of an E state, i.e. these cells are non-convertible (Fig. 5A, blue box). For sufficiently high levels of *S*, cells are able to undergo EMT and produce a range of EMT states, i.e. corresponding to convertible clones (Fig. 5A, red box). From analysis of the EMT transition landscapes at different levels of input stimulus (*S*_*i*_), we can obtain the point at which each population is able to transition out of the E state and undergo EMT. For the unperturbed network, cells first transition at *S*_*u*_=*S*_6_, and the mesenchymal state becomes fully accessible at *S*_7_ (Fig. 5B).

Stochastic simulation of EMT networks constructed with AND versus OR logic show markedly different transition paths, allowing us to design experiments that can infer the EMT network logic that is present in HMLER cells. From a baseline transition point obtained by simulating the unperturbed model (*S*_*u*_=*S*_6_; Fig. 5B), we study how the EMT transition landscape changes as the direct or the indirect ZEB activation rates are perturbed. The dose of stimulus at which cells first transition when the inhibition of miR-200 is increased (increased indirect activation) is recorded as *S*_*x*_. The dose of stimulus at which cells first transition when the direct activation of ZEB is increased is recorded as *S*_*y*_. For models constructed with OR logic, we see that *S*_*x*_=*S*_5_ and *S*_*y*_=*S*_2_, i.e. *S*_*x*_>*S*_*y*_ (Fig. 5C). For models constructed with AND logic, we see that *S*_*x*_=*S*_3_ and *S*_*y*_=*S*_5_, i.e. *S*_*x*_<*S*_*y*_ (Fig. 5D). These inequalities offer the means with which to determine the network logic in live cells: if *S*_*x*_>*S*_*y*_, then the network is constructed with OR logic; if *S*_*x*_<*S*_*y*_, then the network is constructed with AND logic.

The same results are observed (the inequalities are upheld) for comparable simulations of SDE models with additive noise (Fig. S9A-D). We note that these simulations rely on multiplicative noise on the scale of low-moderate. In the case of high multiplicative noise (unlike additive noise), we observe – in line with previous work – that the cell fate landscapes are distorted: the resulting approximate attractor states (represented by mean values of the SDE) change as stochastic fluctuations on the landscape grow in size (Fig. S9E).

Sufficient experimental data to discern between these scenarios can be gathered by subjecting non-convertible HMLER clones to different levels of stimulus via SNAIL or TGFβ under two separate perturbations. The first would be the inhibition of miR-200, which could be achieved by steric blocking; the second would be an amplification of the rate of ZEB activation via SNAIL, which could be achieved by epigenetic regulation or the addition of SNAIL/ZEB-interacting co-factors (such as EGR1 or SP1; Wu et al., 2017). By virtue of the inequalities derived above, knowledge of neither the precise model parameterization nor the precise stimulus levels matching simulations are required for inference. By determining the relative points at which cells become convertible, the network logic can be inferred. Observing EMT earlier, when miR-200 is inhibited rather than when ZEB activation is increased, implies that the network is constructed with AND logic, where signals combine multiplicatively. Observing the converse implies that the network is constructed with OR logic, where signals combine additively.

### The role of logic in EMT during wound healing and ontogenesis

Beyond cancer progression and metastasis, the miR-200-ZEB regulatory circuit regulates EMT during development and tissue regeneration (Nieto et al., 2016). Thus, our results on the impact of logic on this network hold for EMT across all these developmental contexts. However, in these settings, other factors also play significant roles.

In wound healing, dynamic and reversible EMT in keratinocytes is crucial for wound closing and re-epithelialization (Haensel and Dai, 2018). Interactions between ZEB and OVOL1/2 or GRHL2 are essential for both the EMT required for migration and the MET controlling re-epithelialization (Haensel and Dai, 2018; Haensel et al., 2019; Chen et al., 2024). Chen et al. (2024) showed that GRHL2 knockdown induced EMT, while GRHL2 overexpression following EMT induced MET. In light of our results above on miR-200, this sensitivity to reversing EMT with GRHL2 expression suggests the GRN controlling epidermal EMT during wound healing employs multiplicative (AND) logic. The earlier EMT observed with GRHL knockdown (see figure 4A in Chen et al., 2024) is also indicative of AND logic in light of our results on experimental design. To validate if GRHL2-ZEB interactions use AND logic, we propose modifying the scratch assay from Chen et al. (2024) with a higher dose of TGFβ. This would mimic direct activation of ZEB, causing earlier EMT with OR logic. If scratch area decreases with GRHL2 knockdown but not with increased TGFβ, this would confirm AND logic for this EMT subnetwork.

In ontogenesis, neural crest cells produce various cell types through tightly coordinated spatiotemporal dynamics (Leathers and Rogers, 2022). EMT allows cells to delaminate and transform into mesenchymal migratory phenotypes (Zhao et al., 2024). The GRN controlling neural crest EMT has been intensively studied and includes many more transcription factors than the core cancer network (Hovland et al., 2020; Simões-Costa and Bronner, 2015); these include inhibition of Ncad by Snai1/2 and Lmo4, and EMT initiation by FoxD3 with Sox 9/10 and AP2 (Simões-Costa and Bronner, 2015; Rogers et al., 2012). Given the size of the network, many transcription factors have multiple regulators, thus making the network prone to additive versus multiplicative logic effects. Another difference in the neural crest is the intricate spatiotemporal regulation of cell fates; this is in contrast to cancer, where dysregulation relaxes such spatiotemporal constraints. Thus, live cell imaging of EMT in neural crest cells may be best suited to disentangle these spatiotemporal effects in the context of different choices of network logic (Li et al., 2019).

Beyond EMT, a model of neural crest specification based on cyclical fate restriction (Kelsh et al., 2021) has been proposed, described mathematically by a double repressilator, a modified version of a canonical circuit (Elowitz and Leibler, 2000). This model undergoes Hopf or SNIC (saddle node on invariant circle) bifurcations (Farjami et al., 2021), i.e. oscillations can be created from a previously stable steady state, characterizing commitment from multipotent cycling to a lineage-restricted fate. Dawes and Kelsh (2021) showed that AND (but not OR) logic simulations give balanced propensity to each fate before commitment. Testing this requires quantifying cell fate proportions. This could be achieved *in vivo* by single-cell RNA sequencing or through *in vitro* differentiation of neural crest in culture or gastruloids (van den Brink et al., 2014).

These examples show how network logic choice impacts conclusions across developmental systems. While multiple logics might yield similar multistable landscapes, the dynamics under different logic assumptions can vary dramatically.

### The effects of logic control the dynamics of cell fate specification during differentiation of the inner cell mass

In the blastocyst, the inner cell mass (ICM) differentiates into epiblast (Epi) and primitive endoderm (PrE) cells at E3.5 in mice or 5-6 days post-fertilization in humans (Surani et al., 2007). This specification of cell fates can be described by a network consisting of Nanog and Gata6 with regulation from ERK via Fgf4 and/or Fgfr2 (Bessonnard et al., 2014). Analysis of ICM fate decisions has revealed that mixed logic is consistent with data on early embryogenesis in wild type and various Gata6 or Nanog mutant mouse models (Bessonnard et al., 2014). This presents an opportunity for studying the effects of logic in more-complex scenarios than the simpler AND or OR logic considered above. Although it has been suggested that only one logical construction agrees with the data (Mot et al., 2016), we found that multiple logical configurations permitted tristability and investigated the impact of logic choice on cell fate decision making in the ICM.

**Fig. 6.**
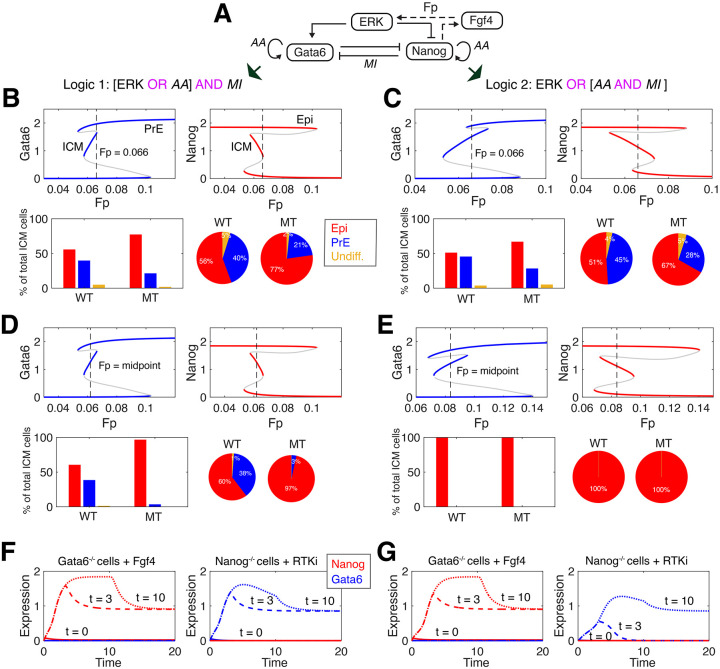
**Alternative mixed logic models can explain cell fate decisions in the ICM.** (A) Schematic of the model: intracellular mutual inhibition (MI) between Gata6 and Nanog, each experiencing auto-activation (AA) and regulation from ERK. Dashed lines indicate the extracellular component: secretion of Fgf4 and activation of ERK via the total extracellular Fgf4 (*Fp*). (B) For logic 1: tristable landscapes for Gata6 (blue; PrE fate) and Nanog (red, Epi fate) with respect to *Fp*. Lower panels: for simulations of 500 ICM cells (*Fp*=0.066), the proportion of cells committing to each fate are shown. Undifferentiated ICM cells (Undiff.) are shown in yellow. (C) For logic 2: tristable landscapes for Gata6 (blue; PrE fate) and Nanog (red, Epi fate), with respect to *F*_*p*_. Lower panels as for panel B with *Fp*=0.066. (D) For logic 1: landscapes for Gata6 and Nanog similar to those in panel B but with external Fgf4 levels at the midpoint of the ICM state (*Fp*=*midpoint*). (E) As for panel C for logic 2. (F) The dynamics of ICM differentiation for logic 1 in Gata6^−/−^ cells with the addition of Fgf4 at different times [*t*∈(0, 3, 10)] (left); or in Nanog^−/−^ cells with the addition of RTKi at different times [*t*∈(0, 3, 10)] (right). (G) As for panel F for logic 2.

The logic with which ICM differentiation was previously modeled (Bessonnard et al., 2014) combines multiplicative mutual inhibition (MI) between Gata6 and Nanog with additive regulation on Gata6/Nanog coming from auto activation (AA) and from ERK (logic 1; Fig. 6A). We found that a second logical configuration, combining multiplicative regulation between AA and MI with external additive regulation from ERK (logic 2; Fig. 6A), also permitted tristability. We simulated ICM differentiation specified by either logic 1 or 2 under the same conditions as were previously studied (see Materials and Methods): with random fluctuations between cells in Fgf4 around an average external value (*Fp*=0.066) that lies within the tristable region of both models (Fig. 6B,C).

Analysis of the proportion of cells differentiating to PrE (Gata6 high) or Epi (Nanog high) fates in the wild-type or Gata6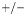 (MT) ICM found that both models exhibited similar qualitative behavior, with approximately equal proportions of PrE and Epi fates in the wild type, and an increase in bias towards the Epi fate in Gata6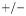 ICM (Fig. 6B,C). Thus, both models recapitulate the experimental observations of Epi/PrE ratios measured in wild-type and Gata6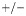 embryos (see figures 3 and 4 in Bessonnard et al., 2014). Stochastic simulations of populations of differentiating ICM cells found similar distributions for logics 1 and 2 (Fig. S10). Comparative analysis of the stochastic dynamics of cell fate commitment (Fig. S10) and the quantitative data presented by Bessonnard et al. (2014) (in figures 3B-E and 5D therein) show that the models recapitulate the earlier specification of Epi fates and the distributions of Nanog/Gata6 expression near steady state, albeit with different sources of noise present in the data compared to simulation.

The Gata6 cell fate landscape for logic 2 (Fig. 6C) is type 1U2D, i.e. the ICM region extends beyond the tristable region. For both PrE and Epi fates to be accessible from any ICM state (a potential requirement for viable embryos), the intermediate ICM state should be entirely contained within the tristable region (1U1D bifurcation type). We investigated an alternative parameterization of the model specified by logic 2 that exhibits 1U1D type tristability, achieved by increasing the threshold for activation of Gata6 by ERK by 70%. For fair comparison of models, we set *Fp* (external Fgf4) to be equal to the midpoint of the tristable region for both logics 1 and 2 (Fig. 6D,E). Under these conditions, the distribution of cells committing to PrE and Epi fates for logic 1 agrees with the data for wild type: in the Gata6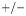 MT, the fraction of PrE cells is lower than expected. For logic 2, however, for both wild type and MT, all cells commit to an Epi fate and no PrE cells are observed. This highlights how – as we saw above for the EMT regulatory network – different logical constructions of the same model can exhibit similar bifurcation landscapes and yet very different dynamics as parameters are perturbed. For both logics, the bifurcation landscapes (Fig. 6D,E) appear consistent with ICM differentiation, yet the dynamics are not.

To emphasize that multiple logics can be consistent with experimental data over a range of conditions, we show that both logics 1 and 2 can recapitulate the dynamics observed in homozygous Gata6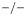 or Nanog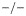 mutants. In Gata6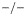 embryos, simulation of ICM differentiation under logic 1 or 2 with the addition of FGF4 at early stages (*t*=0) does not induce Nanog expression, but its addition at later stages (*t*≥3) induces stable Nanog expression (Fig. 6F,G), recapitulating the dynamics observed experimentally in blastocysts by Bessonnard et al. (2014). In Nanog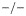 embryos, we analyzed if the addition of a receptor tyrosine kinase inhibitor (RTKi) was able to induce the stable expression of Gata6. For logic 1, addition of RTKi at early stages does not induce Gata6 expression, but its addition at *t*≥3 induces stable Gata6 expression, in agreement with the data and previous simulations (Fig. 6F). For logic 2, addition of RTKi at *t*=3 induces Gata6 expression that is transient and returns to zero; however addition of RTKi at *t*=10 does induce and maintain Gata6 expression (Fig. 6G). The mapping between simulation time and experimental time is not accurately known, thus the delayed stabilizing of Gata6 expression with logic 2 in Nanog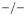 cells can be consistent with the data. Thus, both logical constructions produce dynamics that are consistent with homozygous knockouts. To distinguish between logics and reveal the ICM network wiring, along similar lines to the experimental design for the EMT network above, experiments performed with multiple doses of RTKi at early and/or late timepoints could be used.

To summarize, we have seen here for the ICM network (as for the EMT network above) that caution must be taken in the logical construction of ODE models. Different logics can produce very similar bifurcation landscapes in the unperturbed setting (see the EMT landscape in Fig. 2 or the ICM landscape in Fig. 6D,E) yet when perturbed (by direct and/or indirect activation of ZEB in EMT, or by genotypic perturbations in the ICM) different logical choices give rise to starkly different dynamics. Experiments measuring the relative dynamics of species for perturbed and/or unperturbed networks, if carefully designed, can distinguish between these to determine the logic used by regulatory networks in cell fate decisions.

## DISCUSSION

We have developed a GRN model of EMT to study the effects of network logic on cell state transition paths. The GRN describes the regulation of ZEB by two distinct routes: direct activation of ZEB by SNAIL or indirect activation of ZEB through a double inhibition, i.e. SNAIL⟞miR-200⟞ZEB (Barrallo-Gimeno and Nieto, 2005; Kaufhold and Bonavida, 2014; Sánchez-Tilló et al., 2010; Zhang et al., 2015). The presence of two coupled positive-feedback loops leads to tristable landscapes for this GRN. Through bifurcation and statistical analyses, we have shown that the choice of network logic (multiplicative versus additive) is important: different choices of logic lead to divergent EMT phenotypes. We have also demonstrated how model predictions can be used to design experiments to discriminate between EMT networks, which could thus be used to infer the network logic of the network in live cells.

Current literature characterizing the role of miR-200 in EMT has shown that when miR-200 is inhibited, ZEB transcription factors are upregulated (Korpal et al., 2008; Gregory et al., 2008; Hill et al., 2013; Klicka et al., 2022; Title et al., 2018; Díaz-López et al., 2014). Moreover, re-expression of miR-200 can cause a loss of mesenchymal traits and a reverse MET transition to an epithelial state (Korpal et al., 2008; Nagai et al., 2024; Kong et al., 2009; Zhang et al., 2012), although hypoxia may induce an irreversible EMT (Brown et al., 2024). These data are consistent with EMT network models constructed with multiplicative logic, i.e. where SNAIL and miR-200 act cooperatively to regulate EMT. In agreement with this prediction, recent studies in a mesenchymal cell line have found that the induction of miR-200 cells causes cells to transition into a hybrid E/M state that promotes collective cell migration and expresses epithelial genes (Nagai et al., 2024), although it may be possible to design strategies to avoid hybrid states during MET (Kim et al., 2023).

Divergent EMT phenotypes were observed by perturbing two regulation parameters: the direct activation of ZEB by SNAIL and the inhibition of miR-200 by SNAIL. Unlike the self-activation of ZEB, these two parameters were highly sensitive to the combinatorics of gene regulation. The relevance of these results extends beyond EMT: the network structure of the GRN is found in a variety of biological systems (Mangan and Alon, 2003; Frankhouser et al., 2024). Differentiation of progenitor cells to more differentiated states is often described mathematically by a model undergoing a saddle node bifurcation (of the same type as studied here). Thus, where different regulatory network models of cell differentiation exhibit saddle node bifurcations, our results on the impact of logic are likely relevant, although the exact contributions of different network perturbations will depend on the model.

There are limitations to the current work to be addressed in future studies. The EMT network studied does not explicitly consider the transcriptional species of SNAIL or ZEB, unlike alternative EMT models (Tian et al., 2013). If mRNA species were to be added to the model, the state space would expand to five dimensions, and a simple feedforward loop would no longer be discernible (Fig. S8). Nonetheless, future work ought to consider more-sophisticated regulatory paths.

While a substantial body of literature supports the EMT network considered here (Tian et al., 2013; Zhang et al., 2014; Korpal et al., 2008; Gregory et al., 2008; Hill et al., 2013; Klicka et al., 2022; Title et al., 2018; Díaz-López et al., 2014), additional factors also regulate EMT during cancer and could change the dynamical landscape. Epigenetic factors are important (Al-Radhawi et al., 2022) and alternative models of the EMT network permit tetrastability (Hong et al., 2015; Kim et al., 2023). In such a case, two hybrid E/M states exist, giving rise to six saddle points and, thus, many more possible transition paths. Additional hybrid states have clinical implications: if, on such an EMT landscape, cells can more readily access one or more hybrid states, this could increase metastatic potential. The effects that network logic mediate with larger EMT networks or in light of epigenetic regulatory factors on multistable landscapes will be an important direction for future work.

The core structure of both the EMT network and the ICM differentiation network defines a coherent feed-forward loop. This network motif is found in a variety of biological systems (Mangan et al., 2003; Kalir et al., 2005; Pieters et al., 2021); however, it would be interesting to extend analyses to alternative network motifs of three or four interacting species (Alon, 2020; Mayer et al., 2023). As discussed above, in larger networks it becomes more challenging to distinguish direct versus indirect regulations. Moreover, in larger networks simple AND versus OR logic becomes less likely; the probability of mixed logic will increase with the network size.

The changes in phenotype observed required perturbation of at least two network edges, i.e. a systems biology approach is inherently required (single perturbations are insufficient). This complicates experimentation, although as we outline, it will still be possible to distinguish effects via experiments, e.g. in HMLER cells (Zhang et al., 2022), by first perturbing miR-200 inhibition and then activation of ZEB. Analysis of EMT transition paths required inference of the landscapes from the GRN. Alternative landscape-based approaches do not require knowledge of the specific GRN and offer the means to study transition paths when the GRN is not known (Seyboldt et al., 2022; Boukacem et al., 2024; Liu et al., 2023).

In this work, through the analysis of GRNs controlling cell phenotypes in EMT and development, we have shown how network logic dictates phenotype in subtle yet important ways. In cancer metastasis, this will help us to design interventions that decrease the probability that cells undergo EMT or form hybrid E/M cell states with metastatic potential. In wound healing, we may be able to design more efficient strategies for re-epithelialization. In neural crest development, it may be possible to design GRN perturbations to direct and control cell fates. Overall, we have shown how small logical gene networks can be constructed to achieve desired outcomes in cells upon perturbation, of relevance in synthetic biology and the design of gene networks *in silico*. The ability to explain how multiple signals regulate their target genes offers new means with which to understand the dynamics of gene regulatory networks and the cells fate decisions that they control.

## MATERIALS AND METHODS

### A three-node GRN model of EMT

We study a three-node GRN model that characterizes EMT (Fig. 1B). This network consists of an input signal SNAIL (*S*) that regulates miR-200 (*A*) and ZEB (*B*). We investigate two mathematical models of this network that differ in their network logic.

The model derived from this EMT network constructed with AND logic (given by Eqn 1) is:

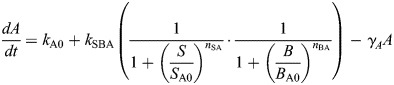


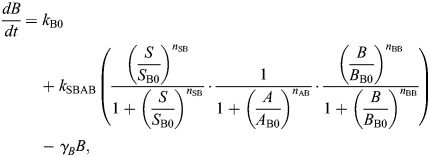
where the parameter descriptions and values used are given in Table 1.

The model derived from this EMT network constructed with OR logic (given by Eqn 2) is:

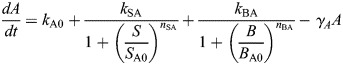


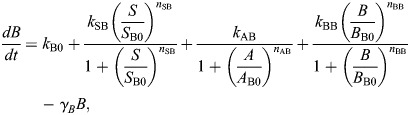
where the parameter descriptions and values used are given in Table 1. The parameters *k*_A0_, *k*_B0_ are associated with the basal synthesis rate of *A* and *B*, respectively. The maximal expression rate of *A* due to *S* and *B* is represented by *k*_SBA_ for the AND model and by *k*_SA_ and *k*_BA_, respectively, for the OR model. The parameters *S*_A0_, *B*_A0_ are associated with threshold values of *S* and *B*, respectively, at half-maximal value of *A*. The Hill coefficients on *A* due to *S* and *B* are given by *n*_SA_ and *n*_BA_, respectively. The maximal expression rate of *B* due to *S*, *A* and *B* is represented by *k*_SBAB_ for the AND model and by *k*_SB_, *k*_AB_ and *k*_BB_, respectively, for the OR model. The parameters *S*_B0_, *A*_B0_ and *B*_B0_ are associated with threshold values of *S*, *A* and *B*, respectively, at half-maximal value of *B*. The Hill coefficients for regulation of *B* via *S*, *A* and *B* are *n*_SB_, *n*_AB_ and *n*_BB_, respectively. The parameters *γ*_*A*_, *γ*_*B*_ represent the degradation of *A* and *B*, respectively.

### Generation of tristable EMT models

To generate many tristable models with either AND or OR logic, we followed the automated bifurcation generator method developed by Dey and Barik (2021), in which the multi-dimensional ODE system is reduced to a one-dimensional ODE using a transfer function.

Briefly, given the ODEs for the AND model:

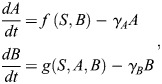
at steady state, 
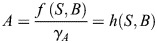
, we have:

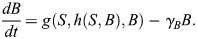


The effective force *F*(*S*, *B*) of the system is thus:

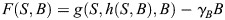
and the effective potential *V*(*S*, *B*) is given by:

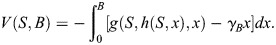


The effective potential is then used to plot potential landscapes for each model. Potential landscapes are plotted in the range *B*∈[0, 3000] and *S*∈[0, 300]; all other parameters were sampled from uniform distributions over the ranges specified in Table 1. For a sampled parameter set, potential landscapes are generated for many values of 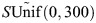, and for each landscape the total number of local minima and maxima are recorded. If at any value of *S* the potential energy landscape contains three local minima and two local maxima, the parameter set is recorded as permitting tristability. Sampling continued until a total of 300 parameter sets permitting tristability were recorded for each of the AND and OR models.

### Perturbation analysis of tristable EMT models

Comparisons of phenotypes on the EMT landscapes were performed by perturbing tristable EMT models in the following manner. Given a parameter set that permits tristability, and the corresponding bifurcation plot (the unperturbed EMT landscape *U*), we investigate the effects of perturbing two parameters: the inhibition by *S* on miR-200 (indirect activation parameter: *S*_A0_) and the direct activation of ZEB by *S* (the direct activation parameter: *S*_B0_), i.e.

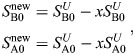
where *S*^*U*^ denotes the unperturbed parameter and we consider perturbations of size *x*=0.25 and *x*=0.5. Note that, due to the form of the model, we refer to a decrease in the parameter value by *x*% as an increase in the strength of regulation via that parameter by *x*%. All other parameters remain unchanged, and we compare the unperturbed landscapes with the landscapes generated by 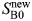 or 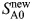.

Perturbation analysis is performed for both the constrained and the unconstrained models (defined in Table 1). For the constrained models, 300 tristable models, as described above, were perturbed and their potential energy landscapes were generated. 150 AND models and 108 OR models permitted tristability following perturbation of both *S*_B0_ and *S*_A0_ by 25%.

### Analysis of EMT landscape properties: SN sensitivity, M state attractor size and bifurcation type

We assess the sensitivity of a saddle node (SN) point to a change in parameter using the mean shift in value of that SN point for a set of landscapes for perturbed models relative to their unperturbed counterparts via:

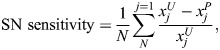
where *x*^*U*^ are the SN point values for unperturbed models and *x*^*P*^ are the SN point values for the perturbed models. *j*=150 models for AND logic; *j*=108 models for OR logic. Each of the four SN points is evaluated in the same way.

The size of attractors for the mesenchymal (M) state is quantified by the length of the M state. The length of the M state is measured for *S* as the distance between SN4 and max(SN1,SN3).

The accessibility to the hybrid state is assessed based on the ability of the model to transit through the hybrid state during EMT and MET. During EMT, the length of the hybrid state available is calculated as (SN3−SN1) and during MET it is calculated as (SN4−SN2). A positive value of (SN3−SN1) indicates accessibility of the hybrid state during EMT, i.e. the landscapes exhibit tristable types 2U1D or 2U2D. Similarly, a positive value of (SN4−SN2) indicates accessibility of the hybrid state during MET, i.e. the landscapes exhibit tristable types 1U2D or 2U2D. Larger positive values indicate greater accessibility to the hybrid state in each case.

Further details on the analysis of EMT landscape properties are given in the supplementary Materials and Methods (sections 1 and 2). We also analyzed how different logical combinations affect transitions across the EMT landscape in the supplementary Materials and Methods (section 3).

### Stochastic simulations of EMT GRN models

Stochastic simulations of EMT network models are performed using a stochastic differential equation (SDE) formulation of each model, i.e. given a general ODE model as 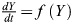 for a vector of species *Y*, we express the stochastic GRN dynamics in the form:

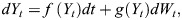
where *f* denotes the EMT network interactions and takes the same form as the ODE model, *g* represents the noise model and *dW*_*t*_ denotes an increment of the Weiner process. The SDE model can be solved numerically using the Euler-Maruyama method, where we assume an Itô interpretation. We consider both multiplicative noise, where the noise model takes the form *g*(*Y*_*t*_)=*σY*_*t*_, with *σ*=0.01 [i.e. noise scales by a constant (*σ*) proportion to the vector of species (*Y*_*t*_)], and additive noise [where the noise model takes the form *g*(*Y*_*t*_)=*σ*, with *σ*=1]. Here the noise is independent of the species *Y*_*t*_.

SDE models constructed with different logic, i.e. with *f*(*Y*_*t*_) specified by the right-hand side of either Eqn 1 (AND logic) or Eqn 2 (OR logic), are simulated for 1000 time steps with a step size of 0.01, which we found appropriate to approximate the stationary state. The final values 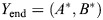 are considered to be the steady-state values. To simulate a population of single cells, we assume that each simulation represents a cell, and consider a population of *N*=1000 cells, i.e. for every value of *S* considered, 1000 trajectories are simulated to obtain a final population of cells 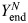. Stochastic simulations are performed at eight values of *S*, i.e. there are a total of 8*N* cells. Steady-state values of *A* and *B* are min-max normalized, i.e. within the total set of 8N simulations:

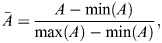
with 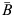 calculated similarly.

### Modeling the differentiation of the inner cell mass

In the model of ICM differentiation introduced by Bessonnard et al. (2014) (see Fig. 6A), Gata6 (*G*) and Nanog (*N*) proteins mutually inhibit each other while also exhibiting auto-activation. ERK signaling via Fgfr2 (*Fr*) enhances Gata6 expression while simultaneously repressing Nanog. Nanog secretes Fgf4; *Fp* is the average level of extracellular Fgf4 perceived by each cell. *Fp* positively regulates intracellular ERK via signaling. Extracellular variability between cells is modeled by randomness in the perceived *Fp* to mimic the heterogeneous distribution of Fgf4 in the local neighborhood of a cell. We followed the implementation of the ICM differentiation model described previously. For full details, see Bessonnard et al. (2014) and Mot et al. (2016). The equations for a single cell under logic 1 ([ERK OR AA] AND MI) are given by:

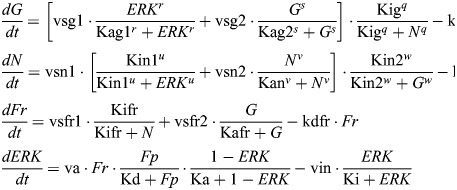
for species [*G*, *N*, *Fr*, *ERK*] and parameters given in Bessonnard et al. (2014). The equations for a single cell assuming the alternative logical construction that we model (logic 2: ERK OR [AA AND MI]) are given by:

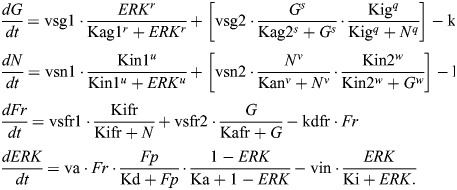


For a single cell, bifurcations are plotted with respect to the control parameter *Fp*. For a population of cells, *Fp* is no longer constant: it becomes a variable that depends on the average extracellular concentration of Fgf4 (*F*). For a N-cell neighbor, 
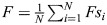
, where *Fs*_*i*_ is the amount of extracellular Fgf4 secreted by the *i*^*th*^ cell and is expressed as:

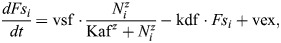
then *Fp* perceived by the *i*^*th*^ cell is given by: *Fp*_*i*_=(1−*γ*_*i*_) · *F*, with 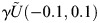 introduced to model the heterogeneity in extracellular Fgf4.

Populations of 25 cells are modeled on a grid, with each cell influenced by its four nearest neighbors (diagonal excluded). The cell fate proportion is then calculated by simulating each cell starting from *G*(*t*=0)=*N*(*t*=0)=0 at time *t*=0 and the cell state recorded in terms of (*G*, *N*) at *t*=60. Cells with high *G*/low *N* are classified as PrE; cells with low *G*/high *N* are classified as Epi. Cells with ambiguous expression of Nanog/Gata6 are classified as undifferentiated.

To simulate ICM differentiation under these alternative logical constructions, we used the same parameter values as previous work (Mot et al., 2016). For the Gata6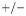 mutant, Gata6 synthesis rates (vsg1, vsg2) were reduced by 15% from wild type. In the wild type, we have vsg1=1.202, vsg2=1; thus, in the mutant, we have vsg1=1.022 and vsg2=0.85. To generate tristable bifurcations of the same type for both logics 1 and 2 (see Fig. 6D,E) one parameter value was changed for logic 2: Kag1 was increased by 70%, i.e. Kag1=0.28+0.7*0.28. In Fig. 6D, *Fp*=0.0619; in Fig. 6E, *Fp*=0.0837. Identical parameter values as in Fig. 6D,E were used to generate the plots in Fig. 6F,G. Bifurcation analysis for the ICM model was performed using the AUTO function in XPPAUT.

Stochastic simulations for the ICM model were performed as described above for the EMT model via SDEs, here assuming multiplicative noise with *σ*=0.1. For each condition, the dynamics of Nanog and Gata6 were tracked and recorded at four time points (*t*=5, 15, 30, 50) for analysis and visualization (scatter plots and pie charts). Cell fates were classified into five groups according to the expression levels of Nanog and Gata6 as follows:

1. Committed PrE (blue): 0≤Nanog≤0.5;Gata6≥1
2. Committed Epi (red): Nanog≥1;0≤Gata6≥0.5
3. PrE-like (cyan): (0≤Nanog≤0.5;0.5≤Gata6≤1) and (0.5≤Nanog≤1;Gata6≤1)
4. Epi-like (magenta): (0.5≤Nanog≤1;0≤Gata6≤0.5) and (Nanog≥1;0.5≤Gata6≤1)
5. Undifferentiated ICM (black): (0≤Nanog≤0.5;0≤Gata6≤0.5) and (0.5≤Nanog≤1;0.5≤Gata6≤1) and (Nanog≥1;Gata6≥1).

## Supplementary Material



10.1242/develop.204583_sup1 Supplementary information
