# Supplementary material for: Transition paths across the epithelial-mesenchymal transition landscape are dictated by network logic
Source: Development. 2025 Jun 12;152(11):dev204583. doi: 10.1242/dev.204583 (PMC12188241; doi:10.1242/dev.204583)
Supplement: Supplementary information [file develop-152-204583-s1.pdf]

Contents

Supplementary Section 1: Analysis of properties of tristable EMT landscapes **2**

Supplementary Section 2: Analysis of network logic for a wide family of models **3**

Supplementary Section 3: Network logic and the combinatorics of gene regulation **4**

Supplementary Figures **6**

Supplementary Section 1: Analysis of properties of tristable EMT landscapes

We studied the properties of tristable models further by analyzing three features of the EMT landscape. The first feature is the saddle node point SN1 (Fig. 1B), SN1 encapsulates the initiation of EMT since EMT is initiated when a transition occurs from the epithelial (E) state into an hybrid (E/M) or mesenchymal (M) state with increasing S. Second, for cells in a mesenchymal state, as S decreases MET is initiated at SN4. The length of the M state is measured as the distance between SN4 and SN1 (or SN3 depending on the tristable response type). We analyze the M state length here, rather than SN4, as it is more informative than SN4 alone regarding the size of the state space for mesenchymal phenotypes. Third, we analyze the accessibility of the hybrid E/M state via measuring the distance between the saddle-node points SN3 and SN1 ($SN3 - SN1$) during EMT and measuring $SN4 - SN2$ during MET. A positive value of $SN3 - SN1$ indicate the hybrid state is accessible through the tristable types 2U1D or 2U2D during EMT. Likewise a positive value of $SN4 - SN2$ indicate the hybrid state is accessible through the tristable types 1U2D or 2U2D during MET. The greater the magnitude of the $SN3 - SN1$, the greater the accessibility to the hybrid state during EMT. Similarly, the greater is the magnitude of $SN4 - SN2$, the greater is the accessibility to the hybrid state during MET.

We analyzed the set of models that permitted tristability after perturbations (150 AND models and 108 OR models) as discussed above. For SN1 (the initiation point of EMT), we saw that increasing the indirect activation strength (i.e. increasing the inhibition of miR200) lowered SN1 (the EMT initiation point) in 97% of the AND models tested (Fig. 4A). I.e. the E state is destabilized and EMT is initiated earlier for increases in the indirect activation relative to the direct activation. For OR models we saw the opposite effect: in 98% of models, SN1 is lowered (EMT initiated earlier) when the *direct* activation strength was increased (Fig. 4B). Thus, network logic dictates the relative importance of regulations on ZEB in initiating EMT. For AND logic, the EMT initiation point is more sensitive to the indirect activation (the inhibition of miR-200 by S) than to the direct activation of ZEB. For OR logic, i.e. additive regulation of ZEB, the converse is true: the EMT initiation point is more sensitive to the direct activation of ZEB than to the inhibition of miR-200. In this case, inhibition on miR-200 alone cannot destabilize the E state and initiate EMT without additional direct activation of ZEB.

In a similar manner to SN1, we studied how the size of the mesenchymal state attractor (the length of the M state) changed as direct/indirect activation strengths were perturbed for AND and OR models. Here, we found that for AND models increasing the strength of the indirect activation rate decreased the size of the M state (Fig. 4C). I.e. as the inhibition on miR-200 from S is reduced, the M state is destabilized and cells in the mesenchymal state more readily undergo MET. This is supported by experimental evidence: miR-200 is known to maintain the E state such that suppressing is sufficient to initiate EMT, and re-expression it is sufficient to induce MET (Korpal et al., 2008; Nagai et al., 2024). Analysis of OR models showed the opposite behaviour: increasing the indirect activation (reducing the suppression on miR-200) increased the length of the M state (Fig. 4D), i.e. increasing the likely population size of mesenchymal cells.

We also analyzed how accessibility of the hybrid state was affected by perturbing the direct and indirect regulation rates. For AND models, during EMT, around 55% of the models showed an increased value of $SN3 - SN1$ when the indirect activation regulation was increased by 25% whereas around 45% of the models showed increased value in $SN3 - SN1$ when the direct regulation was increased by 25% (Fig. 4E, left). This suggest similar degree of accessibility to the hybrid state during EMT and MET when direct/indirect regulation was perturbed by 25%. On the other hand during MET, increasing indirect regulation by 25% increased the length of $SN4 - SN2$ in all the AND models thus providing a greater accessibility to the hybrid E/M states (Fig. 4E, right). A

Development • Supplementary information

similar analysis for the OR models showed that when the direct regulation was increased by 25%, around 93% of the models showed increased accessibility of the hybrid state during EMT (Fig. 4F, left) and around 82% of the models showed increased accessibility to the hybrid state during MET (Fig. 4F, right). The observation for AND models is supported by experimental studies where, in MDA-MB-231 cells, it was shown that the induction of miR-200 promoted cells to enter into a hybrid E/M state displaying a hybrid phenotype characterized by collective cell migration and epithelial gene expression (Nagai et al., 2024).

Similar results were obtained when the direct/indirect regulation parameters were perturbed by larger amounts: 50% from the baseline (Fig. S1). We also tested an alternate indirect model parameter on EMT phenotypes. Two interactions in the GRN combine to define the "indirect activation of ZEB:" the inhibition from S to A (S_{A0}; explored above), and the interaction from A to B (A_{B0}). Perturbation analysis performed for A_{B0} generated similar qualitative results for AND and OR models as seen above: where the logic dictates which network perturbation will control specific EMT phenotypes (Fig. S2).

In summary, through perturbation of direct/indirect regulation parameters, alternate EMT states are stabilized or destabilized based on the choice of network logic. For AND models, increasing the inhibition on miR-200 (thus increasing the indirect activation of ZEB) destabilized the accessibility of the epithelial &mesenchymal states and increased the accessibility of the hybrid state. In contrast, for OR models, increasing the inhibition on miR-200 stabilized the epithelial &mesenchymal states and delayed the initiation of EMT. The hybrid E/M state was in this case less accessible. Overall, we saw that when the E state is destabilized, so does the M state and the accessibility of the hybrid state increases, and vice versa. A considerable body of literature on the role of miR-200 in the initiation of EMT and the reverse MET suggests that suppressing miR-200 is sufficient to initiate EMT, and that re-expressing miR-200 in mesenchymal state cells can initiate MET (Kong et al., 2009; Korpal et al., 2008; Nagai et al., 2024; Zhang et al., 2012). In light of these experimental studies and our results on the alternative phenotypes observed with network logic, we predict that the GRN characterizing EMT via regulation of ZEB by miR-200 and SNAIL is constructed with AND logic.

Supplementary Section 2: Analysis of network logic for a wide family of models

Here we analyzed various perturbations to the EMT network model to assess the generality of our results on the impact of logic. First, we relax the assumptions of the "constrained" model in which parameters are set to be equal (see main Methods). Instead, we considered a wide range of parameterized EMT network models that permitted tristability (Table 1 in main text; "unconstrained models"). For models constructed with either AND or OR logic, we sampled parameters and analyzed properties of the EMT landscape.

Through systematic perturbations of the direct and indirect regulations on ZEB for unconstrained models, we observed the same qualitative results as obtained above for constrained models. The EMT initiation point, stability of the mesenchymal state, and accessibility of the hybrid state all exhibit sensitivity to the direct/indirect regulation parameters that is dictated by the network logic (Fig. S3).

We also analyzed the parameters of a different gene regulatory network model of EMT. The ternary chimera switch model (Lu et al., 2013) is constructed with AND logic and has a similar form to the original model (Eqs. 1 in main text) but with a larger number of parameters. For the Lu et al. model, we analyzed the bifurcation landscapes for ZEB mRNA and ZEB protein with respect to SNAIL as the direct or indirect activation strengths were increased by 55.5% (Fig. S4). In agreement with the analysis of AND models above, increasing the indirect activation strength

Development • Supplementary information

in the Lu et al. model lowered the initiation point for EMT, increased the accessibility of the E/M state, and destabilized the M state (Fig. S4). We sought to perform a similar analysis for the OR logic model of Tian et al. (2013), characterized by two cascading bistable switches, however in this model different modules of the network control different parts of the cascading bistable switches. There is therefore no obvious way in which direct vs. indirect regulations of an EMT initiating factor can be defined.

We also investigated the impact of other model parameters on the EMT GRN. Perturbation analysis for the self-activation of B (B_{B0}) and the inhibition on A from B (B_{A0}) was performed for constrained models, revealing that increasing the self-activation of B (decreasing B_{B0}) increased the length of the E/M state in all 150 tristable AND models (Fig. S5A). This observation is in agreement with previous work (Lu et al., 2013), and indeed is a general phenomenon observed for the self-activation rate in tristable networks of this type (Jia et al., 2017). For OR models, in the majority (67%) of cases we saw that increasing the self-activation of B (decreasing B_{B0}) increased the stability of the E/M state (Fig. S5B). For both AND and OR models, increasing self-activation (decreasing B_{B0}) increased the accessibility of the hybrid E/M state (Fig. S5C-D). SN1 also behaved similarly with respect to B_{B0} for AND and OR models (Fig. S5E-F). For the inhibition on A from B (B_{A0}): decreasing the inhibition strength led to increased accessibility/stability of the hybrid state for both AND and OR models (Fig. S6). This demonstrates that — unlike for the direct/indirect activation rates of ZEB — perturbing the self-activation rate of B or the inhibition on A from B leads to consistent phenotypes regardless of the network logic with which the model is constructed. Given the diverging effects that AND vs. OR logic have on the EMT network, we analyzed how multiple inputs combine differentially to produce these results (details in Supplementary Text 2 and Figs. S7) We also considered an expanded network incorporating mRNA explicitly into the model (Fig. S8).

Supplementary Section 3: Network logic and the combinatorics of gene regulation

In light of the opposing effects of network logic on EMT phenotypes observed for a wide variety of tristable models, we explored how different responses to the same parameter perturbations could be explained in terms of combinatorial gene regulation. The node B (ZEB in the EMT model) receives in total three inputs: self-activation, activation from S (direct activation), and inhibition from A (indirect activation by S). For AND models, input regulations to B combine multiplicatively. This implies a strong dependence: the direct activation of B only weakly affects EMT initiation when there remains inhibition on B from A. Thus for AND models, B is less sensitive to direct activation from S. In constrast, increasing the indirect activation rate weakens the inhibition from A on B. This implies a lower activation threshold of B, making it more sensitive to changes in S (earlier initiation of EMT). Increasing the indirect activation strength also destabilizes the M state, thus decreasing the EMT window: the length between SN1 and SN4, i.e. total size of the multistability region (Fig. S7A-B).

For OR models, input regulations to B combine additively, such that each individually regulates B. This relative independence means that even if one of the regulations is low/off, B can still be expressed. In this case, increasing the direct activation rate has a strong effect on B, since S can activate B even in the presence of inhibition from A. Earlier initiation of EMT (lower SN1) reduces the distance between SN1 and SN4, thus reducing the EMT window. For the OR model, increasing the indirect activation rate (less inhibition from A to B) has a weak effect on B, since an increase in S (direct activation) is still required to initiate EMT. Since in this case B is less sensitive to S, also for the reverse MET a greater reduction in S is required to initiate the transition. Thus

Development • Supplementary information

increasing the indirect activation rate increases the EMT window (Fig. S7C-D).

By teasing apart the combinatorial effects of additive vs. multiplicative regulation, the relative importance of specific model parameters can thus be deduced. In the literature, there is evidence that enhancers primarily combine additively (Long et al., 2016; Martinez-Ara et al., 2024), although transcription factors regulate their targets both additively and multiplicatively (Sanford et al., 2020). Here we emphasize that only by analyzing small, core network motifs can these regulations be reliably distinguished. E.g., even in a slightly larger EMT network including ZEB mRNA and protein species (Tian et al., 2013), it is no longer possible to define a direct ZEB activation and thus clearly distinguish between logics. However, by considering the two possible paths we can see that the path closer to direct activation (via ZEB mRNA) behaves similarly to the results above, albeit less distinctly: AND and OR phenotypes show more overlap (Fig. S8).

References

Jia, Dongya, Mohit Kumar Jolly, William Harrison, Marcelo Boareto, Eshel Ben-Jacob, and Herbert Levine (2017). "Operating Principles of Tristable Circuits Regulating Cellular Differentiation." *Physical Biology* 14.3. ISSN: 1478-3975. DOI: 10.1088/1478-3975/aa6f90.

Kong, Dejuan, Yiwei Li, Zhiwei Wang, Sanjeev Banerjee, Aamir Ahmad, Hyeong-Reh Choi Kim, and Fazlul H. Sarkar (2009). "miR-200 Regulates PDGF-D-Mediated Epithelial–Mesenchymal Transition, Adhesion, and Invasion of Prostate Cancer Cells." *Stem Cells* 27.8. ISSN: 1066-5099. DOI: 10.1002/stem.101.

Korpal, Manav, Esther S. Lee, Guohong Hu, and Yibin Kang (2008). "The miR-200 Family Inhibits Epithelial-Mesenchymal Transition and Cancer Cell Migration by Direct Targeting of E-cadherin Transcriptional Repressors ZEB1 and ZEB2*." *Journal of Biological Chemistry* 283.22. ISSN: 0021-9258, 1083-351X. DOI: 10.1074/jbc.C800074200.

Long, Hannah K., Sara L. Prescott, and Joanna Wysocka (2016). "Ever-Changing Landscapes: Transcriptional Enhancers in Development and Evolution." *Cell* 167.5. ISSN: 0092-8674. DOI: 10.1016/j.cell.2016.09.018.

Lu, Mingyang, Mohit Kumar Jolly, Herbert Levine, José N Onuchic, and Eshel Ben-Jacob (2013). "MicroRNA-based Regulation of Epithelial-Hybrid-Mesenchymal Fate Determination." *Proceedings of the National Academy of Sciences* 110.45. DOI: 10.1073/pnas.1318192110.

Martinez-Ara, Miguel, Federico Comoglio, and Bas van Steensel (2024). "Large-Scale Analysis of the Integration of Enhancer-Enhancer Signals by Promoters." *eLife* 12. ISSN: 2050-084X. DOI: 10.7554/eLife.91994.

Nagai, Tomoaki, Misa Sato, and Michiru Nishita (2024). "miR-200c-141 Induces a Hybrid E/M State and Promotes Collective Cell Migration in MDA-MB-231 Cells." *Biochemical and Biophysical Research Communications* 709. ISSN: 0006-291X. DOI: 10.1016/j.bbrc.2024.149829.

Sanford, Eric M, Benjamin L Emert, Allison Coté, and Arjun Raj (2020). "Gene Regulation Gravitates toward Either Addition or Multiplication When Combining the Effects of Two Signals." *eLife* 9. ISSN: 2050-084X. DOI: 10.7554/eLife.59388.

Tian, Xiao-Jun, Hang Zhang, and Jianhua Xing (2013). "Coupled Reversible and Irreversible Bistable Switches Underlying TGFβ-induced Epithelial to Mesenchymal Transition." *Biophysical Journal* 105.4. ISSN: 0006-3495. DOI: 10.1016/j.bpj.2013.07.011.

Zhang, Zhe, Ze-Bing Liu, Wei-Min Ren, Xuan-Guang Ye, and You-Yuan Zhang (2012). "The miR-200 Family Regulates the Epithelial-Mesenchymal Transition Induced by EGF/EGFR in Anaplastic Thyroid Cancer Cells." *International Journal of Molecular Medicine* 30.4. ISSN: 1791-244X. DOI: 10.3892/ijmm.2012.1059.

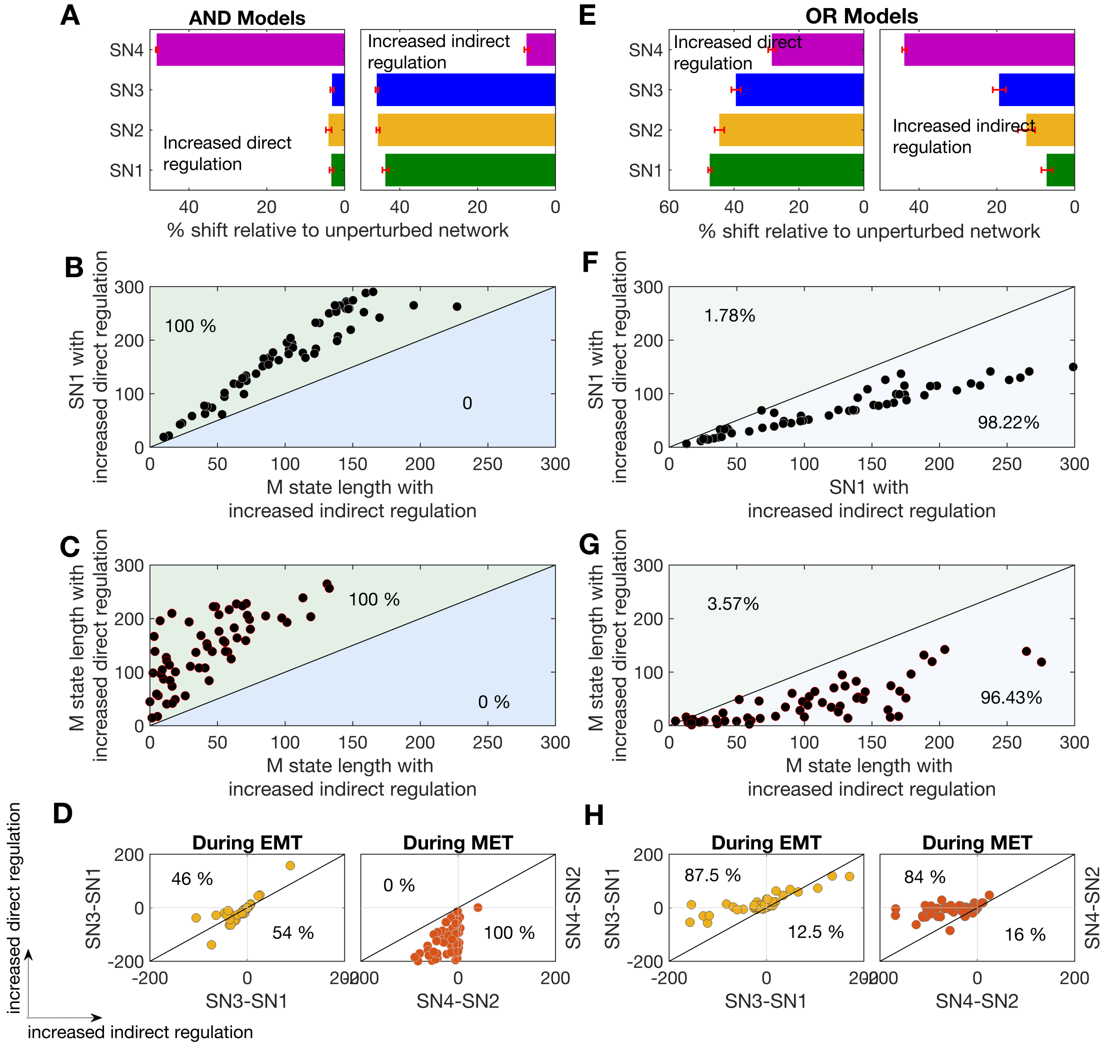

Fig. S1. Impact of varying regulation parameters by 50% in AND/OR models. A,**E.** Sensitivity of the SN points for tristable (**A**) AND models and for the (**E**) OR models perturbed by 50% increase in the direct or the indirect activation strength. **B,F.** Scatter plots of SN1 (the EMT initiation point) for the 50% perturbations to (**B**) AND models and for the (**F**) OR models. **C,G.** Scatter plots for the M state length for 50% perturbations in the (**C**) AND models and for the (**G**) OR models. **D,H.** The accessibility of the hybrid states during EMT and MET for the tristable (**D**) AND models and for the (**H**) OR models.

Development • Supplementary information

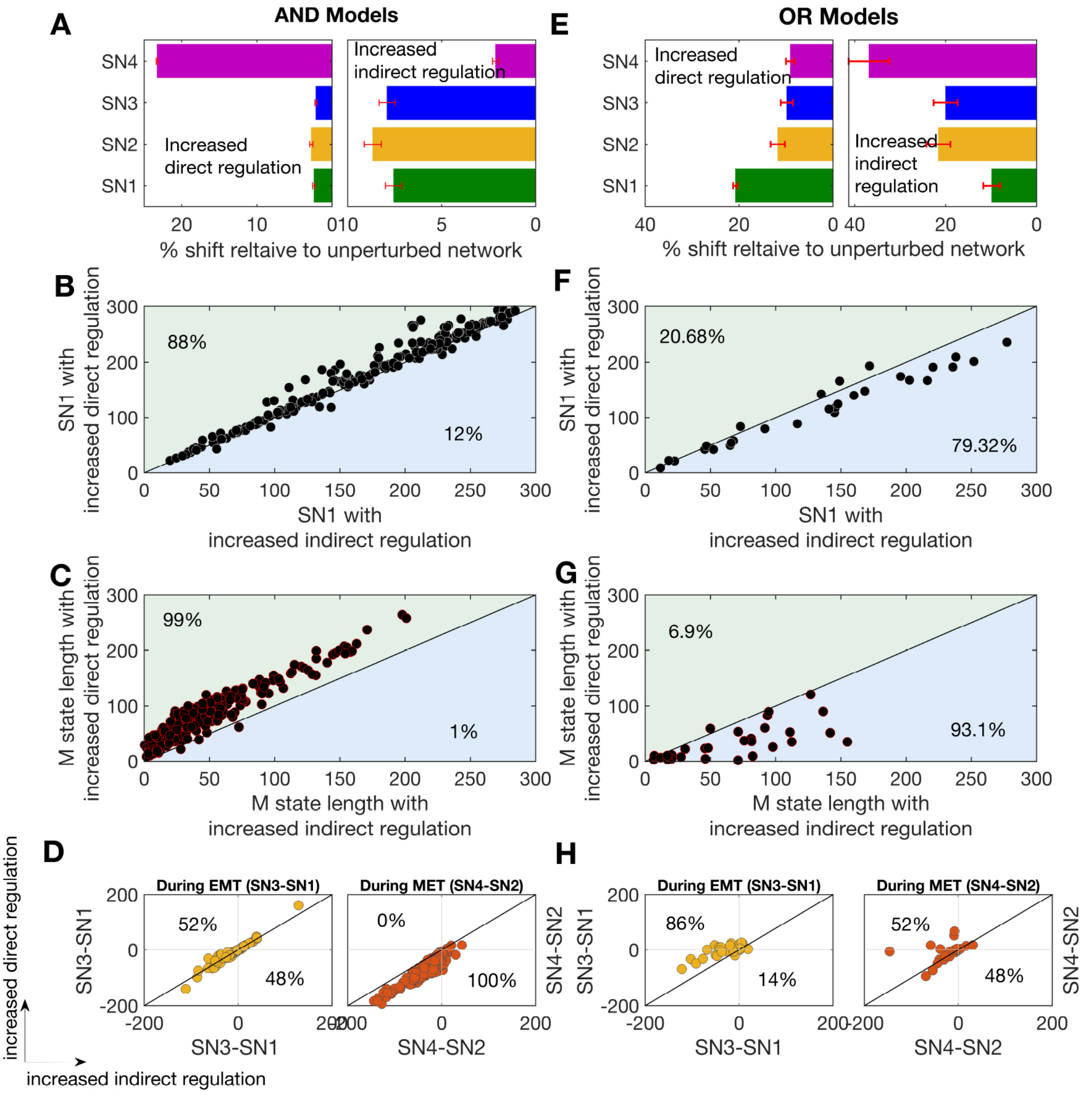

Fig. S2. Impact of varying alternative indirect regulation parameter A_{B0} by 25% in AND/OR models. A,E. Sensitivity of the SN points for tristable (**A**) AND models and for the (**E**) OR models perturbed by a 25% increase in the direct activation strength or the alternative indirect activation strength A_{B0}. **B,F.** Scatter plots of SN1 (the EMT initiation point) for the 25% perturbations to (**B**) AND models and for the (**F**) OR models. **C,G.** Scatter plots for the M state length for 25% perturbations in the (**C**) AND models and for the (**G**) OR models. **D,H.** The accessibility of the hybrid states during EMT and MET for the tristable (**D**) AND models and for the (**H**) OR models.

Development • Supplementary information

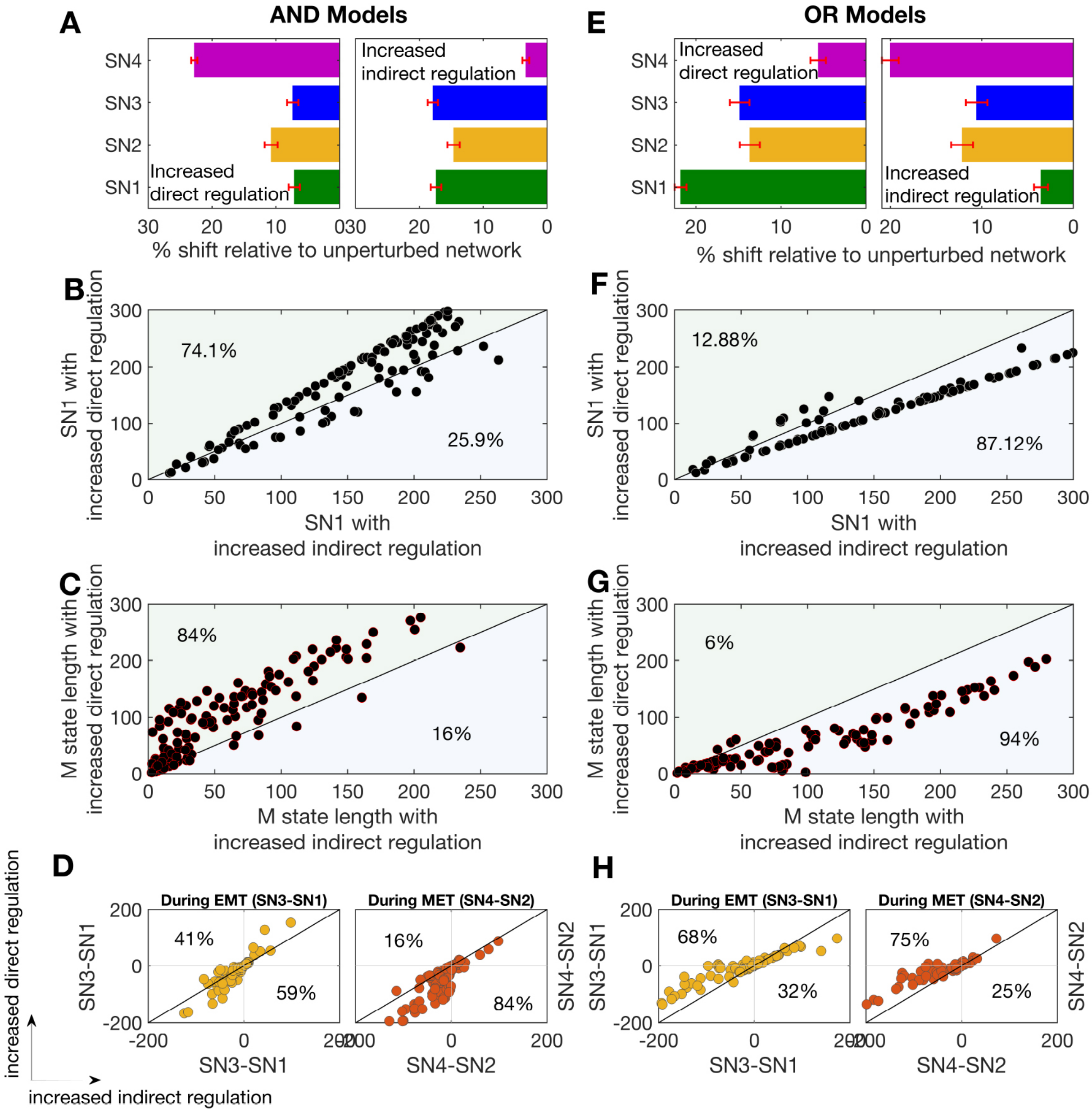

Fig. S3. Impact of varying regulation parameters by 25% for the unconstrained AND/OR models. A,E. Sensitivity of the SN points for the unconstrained tristable (**A**) AND models and for the (**E**) OR models perturbed by 25% increase in the direct or the indirect activation strength. **B,F.** Scatter plots of SN1 (the EMT initiation point) for the 25% perturbations to (**B**) unconstrained AND models and for the (**F**) unconstrained OR models. **C,G.** Scatter plots for the M state length for 25% perturbations in the unconstrained (**C**) AND models and for the (**G**) OR models. **D,H.** The accessibility of the hybrid states during EMT and MET for the tristable (**D**) AND models and for the (**H**) OR models.

Development • Supplementary information

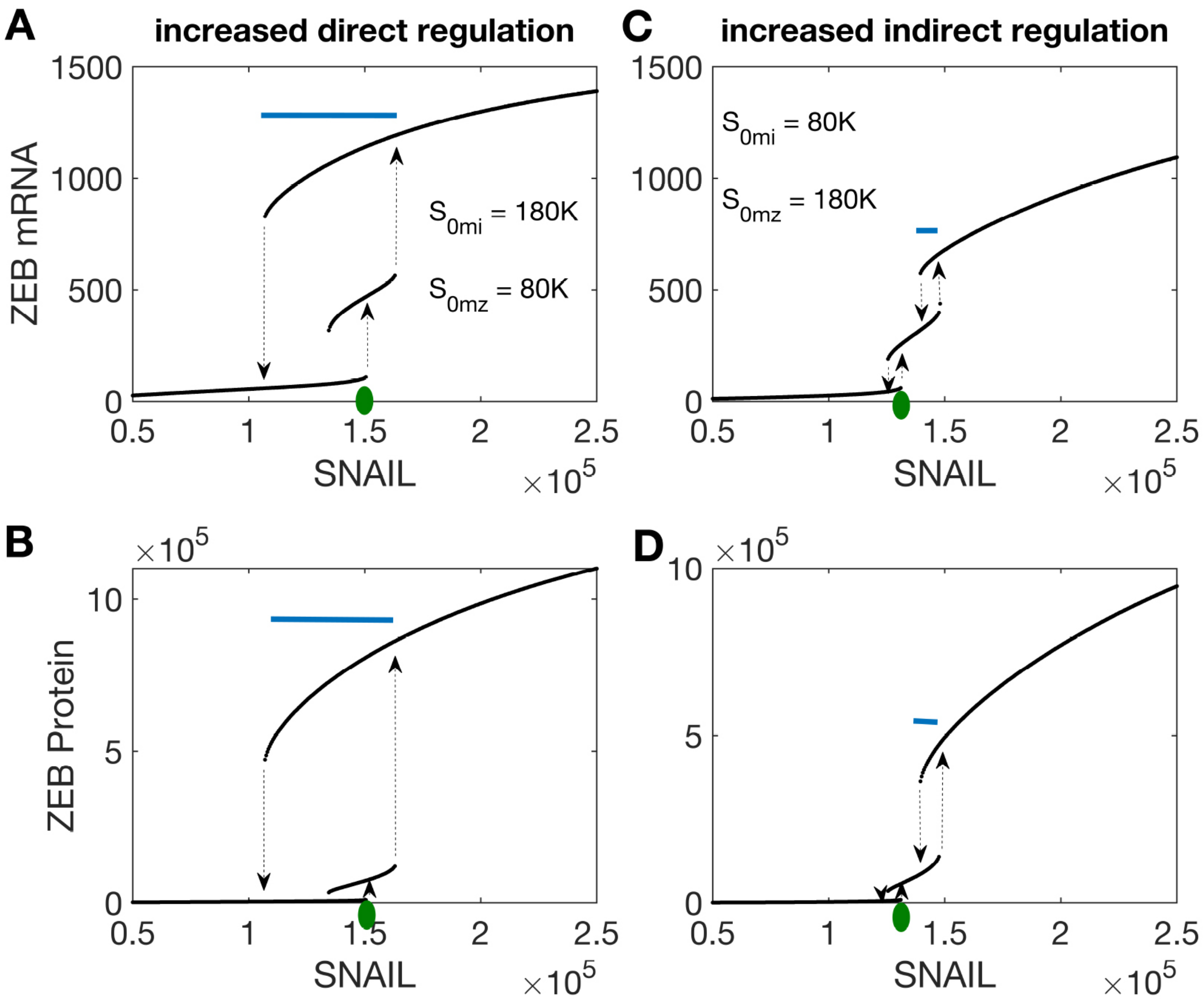

Fig. S4. Analysis of the effects of direct vs indirect regulation parameters in the AND logic model of Lu et al. (2013). A. ZEB mRNA responses to SNAIL as the direct regulation parameter is increased by 55.5%. Initiation point of EMT is indicated by green circle and the length of M state by the blue horizontal bar. **B.** ZEB protein responses to SNAIL as the direct regulation parameter is increased by 55.5%. **C.** ZEB mRNA responses to SNAIL as the indirect regulation parameter is increased by 55.5%. **D.** ZEB protein responses to SNAIL as the indirect regulation parameter is increased by 55.5%.

Development • Supplementary information

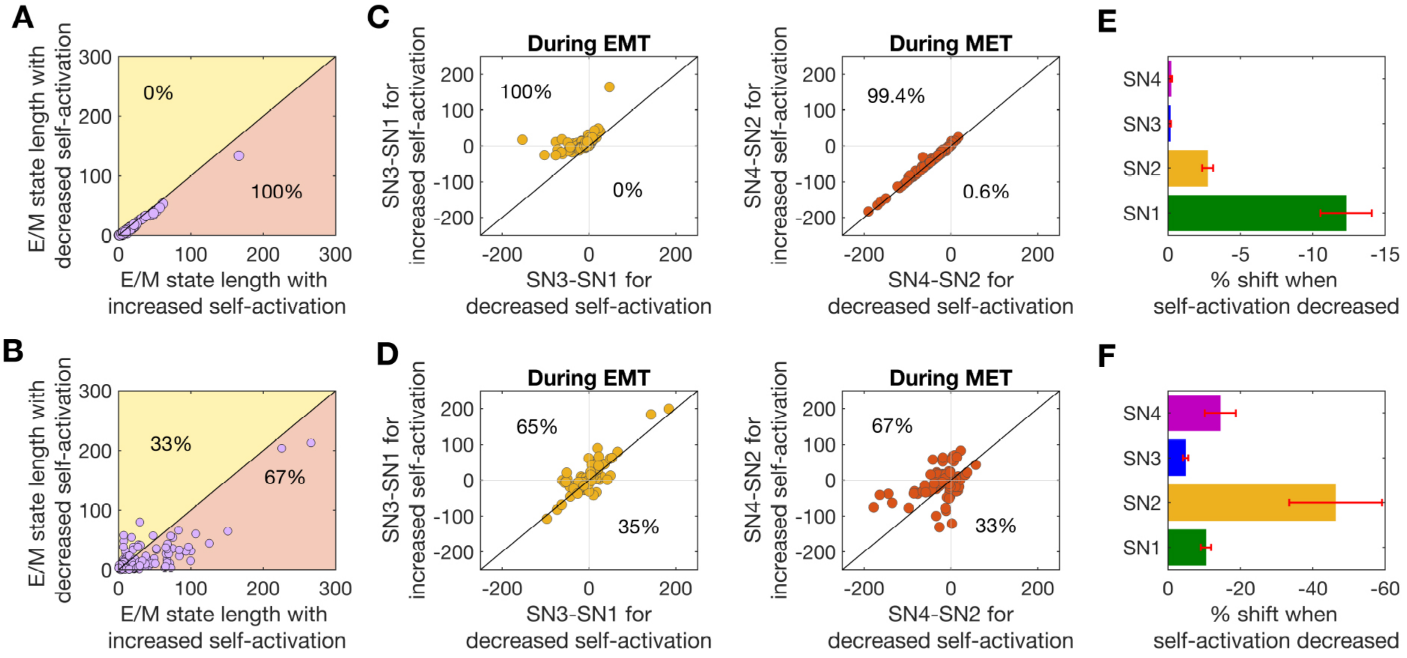

Fig. S5. Analysis of the effects of self-activation parameter B_{B0} in the AND and OR models. A-B. Scatter plot of the length of the hybrid state (measured as $SN3-SN2$) for 25% perturbations in AND models (**A**) and in OR models (**B**). **C-D.** Accessibility of the hybrid state for the 25% perturbation in the self-regulation during EMT and MET for the AND models (**C**) and OR models (**D**). **E-F.** Sensitivity of the SN points for the 25% perturbations in the self-activation parameter for the AND models (**E**) and OR models (**F**).

Development • Supplementary information

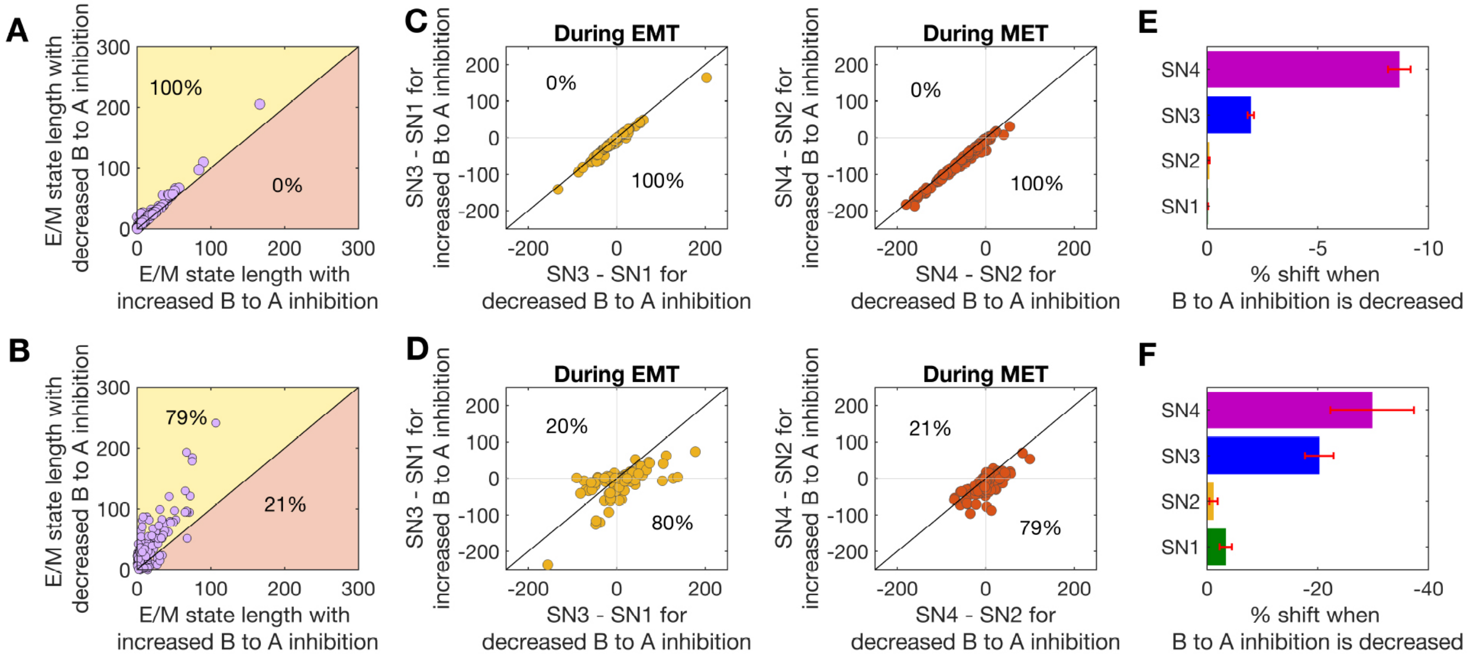

Fig. S6. Analysis of the effects of inhibition parameter B_{A0} in the AND and OR models. A-B. Scatter plot of the length of the hybrid state (measured as $SN3 - SN2$) for 25% perturbations in AND models (**A**) and in OR models (**B**). **C-D.** Accessibility of the hybrid state for the 25% perturbation in B_{A0} during EMT and MET for the AND models (**C**) and OR models (**D**). **E-F.** Sensitivity of the SN points for the 25% perturbations in B_{A0} for the AND models (**E**) and OR models (**F**).

Development • Supplementary information

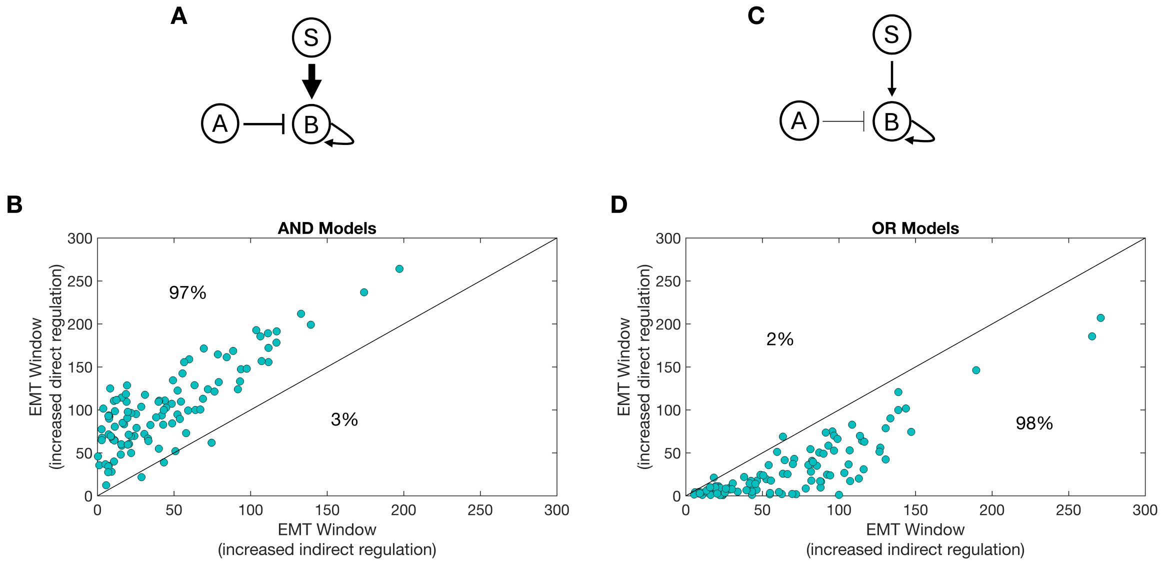

Fig. S7. Analysis of the combinatorial impact of network logic on EMT landscape features. A,B Scatter plot of the size of the EMT window (distance from SN1 to SN4) for 25% perturbations to the direct vs indirect regulation parameters for the 150 AND models. **C,D.** Scatter plot of the size of the EMT window (distance fron SN1 to SN4) for perturbations to the direct vs indirect regulation parameters for the 108 OR models

Development • Supplementary information

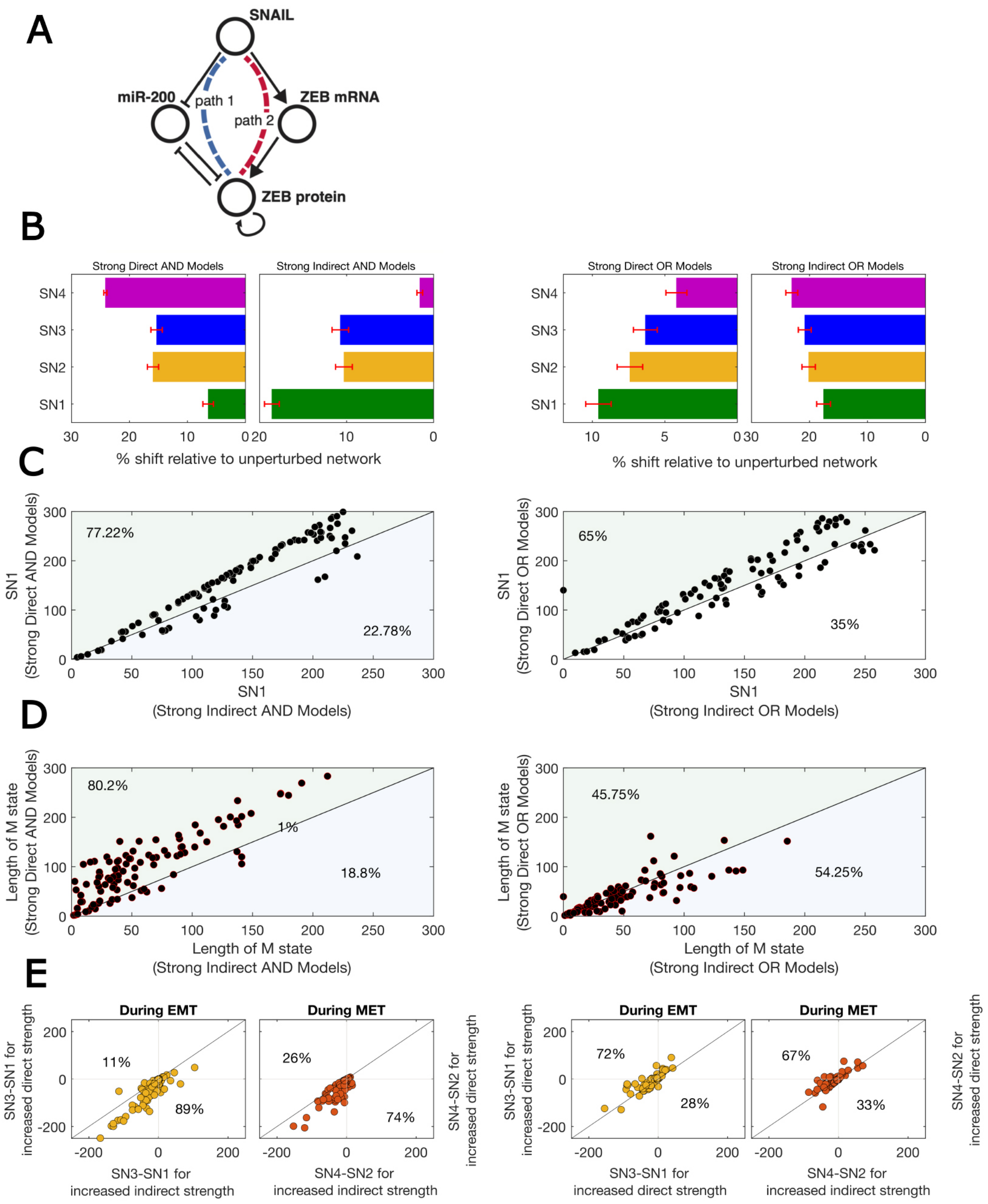

Fig. S8. Features of EMT landscapes for AND vs OR models incorporating mRNA. A. Schematic of the EMT network models that incorporates both mRNA and protein species of ZEB. Path 1 is analogous to indirect regulation in previous models; path 2 is analogous to direct reg-ulation. **B.** Sensitivity of SN points for the EMT networks perturbed by 25% increase in path2 (indirect) or path 1 (direct) regulation stregth for AND models (left) and OR models (right). **C.** Scatter plots of SN1 (the EMT initiation point) for the perturbed AND models (left) and OR models (right). **D.** Scatter plot measuring the length of M state for the pertubed AND models (left) and OR models (right). **E.** The accessibility to the hybrid states during EMT and MET for the perturbed AND models (left) and OR models (right).

Development • Supplementary information

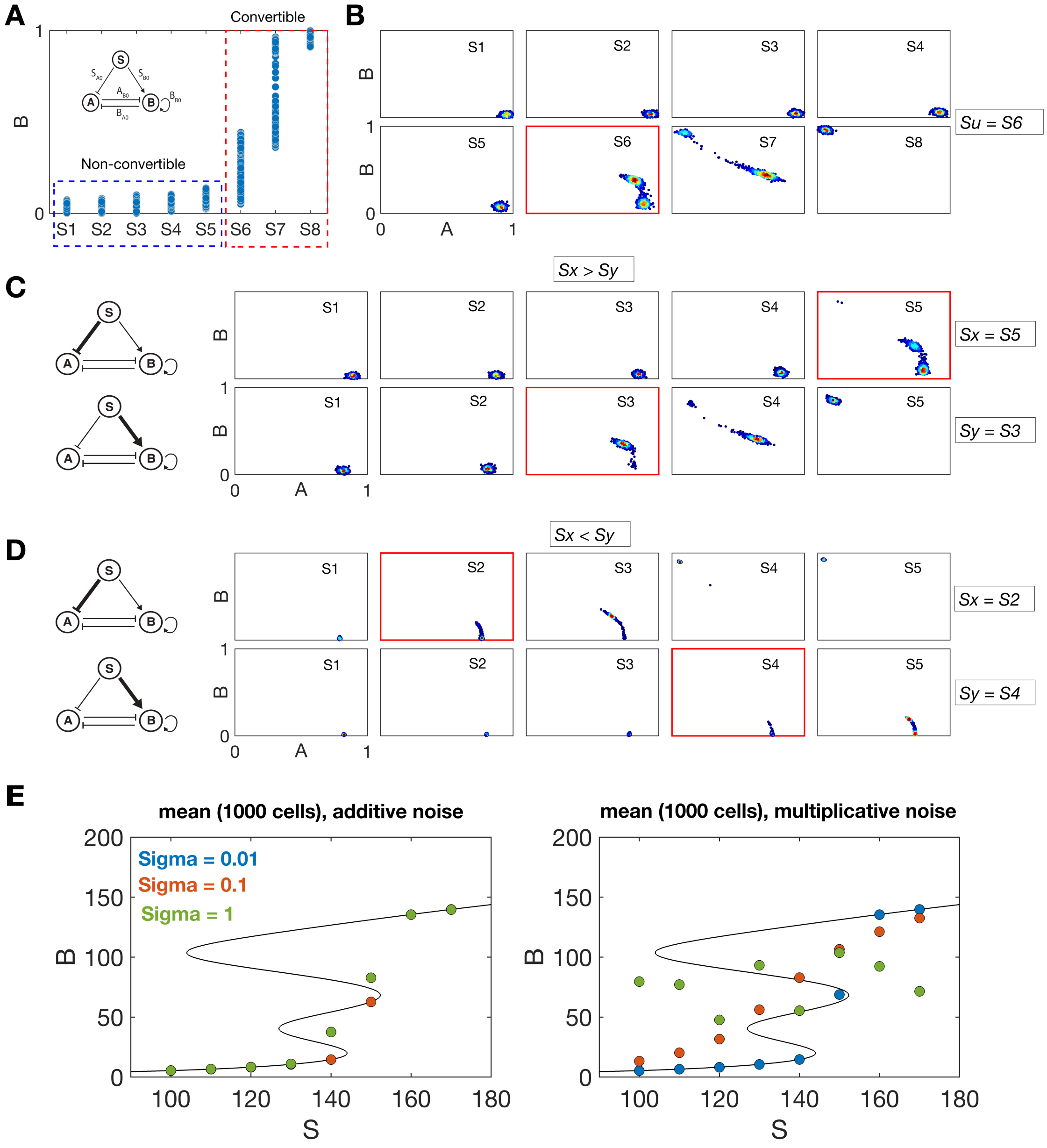

Fig. S9. Experimental design reveals the network logic of GRNs for EMT. A. Stochastic simulations of the EMT GRN network using additive noise for different S values can categorize cells as non-convertible (signal levels $S = S_1$–S_5; blue box) or convertible (signal levels $S \geq S_6$; red box). **B** Equilibria of the model in the (A, B) phase plane for stochastic differential equation simulations of the unperturbed model. S_i indicates an input signal strength of signal S. **C.** Equilibria of the model in the (A, B) phase plane for simulations of the OR logic GRN. Either the indirect (top row) or the direct (bottom row) regulation on ZEB is perturbed and the minimum signal required to initiate EMT is recorded (S_x for indirect and S_y for direct). **D.** Equilibria of the model in the (A, B) phase plane for simulations of the AND logic GRN, perturbing either the indirect or the direct regulation as for (C). **E.** Mean value of 1000 cells at different 'S' for different noise strengths for the SDE calculated using additive noise (left) and multiplicative noise (right).For additive noise, the mean values coincides with the deterministic stable points for different noise strengths whereas for the multiplicative noise, the mean values shift away from the deterministic stable points as the noise strtength increases.

Development • Supplementary information

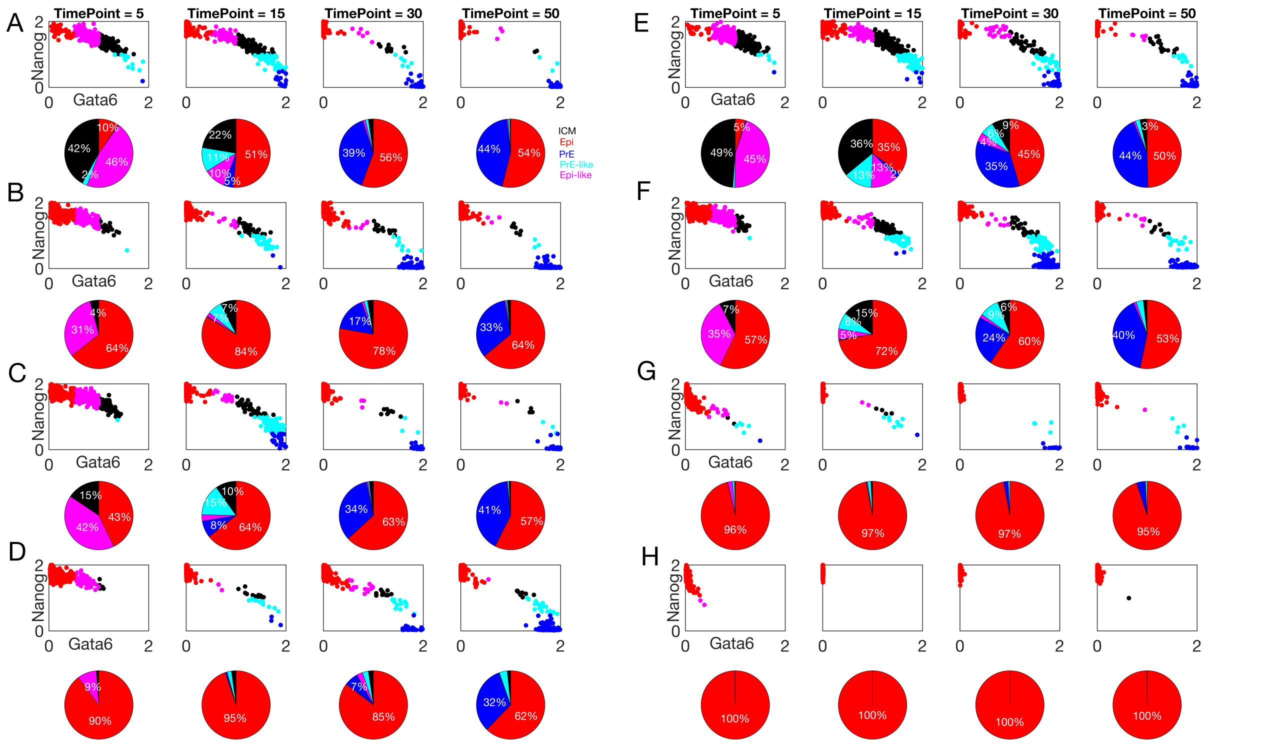

Fig. S10. Stochastic simulations for the ICM cell differentiation for two logics in Fig. 6A. A,E. Stochastic simulations for the tristable bifurcation generated by the same parameter values in both the logics with average Fp = 0.066. Snapshots taken at 4 different time points and the proportion of cells are calculated (shown in pie charts) for the Logic 1 (**A**) and for Logic 2 (**E**) for wild type (WT). **B,F**. Similar calculations were done for the mutant (MT) types in both the logics. **C,G**. Stochastic simulations and cell proportions for the adjusted tristable bifurcation in Logic 2 and unchanged tristable for Logic 1 with the average Fp value at the centre of the respectable tristable region for the WT for Logic 1 (**C**) and for Logic 2 (**G**). **D,H**. Stochastic calculations for the above conditions for MT for Logic 1 (**D**) and Logic 2 (**H**).

Development • Supplementary information
