# Supplementary material for: Transition paths across the epithelial-mesenchymal transition landscape are dictated by network logic
Source: Development. 2025 Jun 12;152(11):dev204583. doi: 10.1242/dev.204583 (PMC12188241; doi:10.1242/dev.204583)
Supplement: Supplementary file 1 [file develop-152-204583_review_history.pdf]

Transition paths across the epithelial-mesenchymal transition landscape are dictated by network logic

Anupam Dey and Adam L. MacLean
DOI: 10.1242/dev.204583

Editor: Paul François

Review timeline

Original submission:	6 December 2024
Editorial decision:	3 February 2025
First revision received:	29 April 2025
Accepted:	12 May 2025

Original submission

First decision letter

MS ID#: dev.204583

MS TITLE: Transition paths across the EMT landscape are dictated by network logic

AUTHORS: Anupam Dey and Adam L. MacLean

Dear Adam

I have now received all the referees' reports on the above manuscript, and have reached a decision. The referees' comments are appended below, or you can access them online: please go to:

As you will see, the referees express considerable interest in your work, but have some significant criticisms and recommend a substantial revision of your manuscript before we can consider publication. If you are able to revise the manuscript along the lines suggested, which may involve further experiments, I will be happy receive a revised version of the manuscript. Your revised paper will be re-reviewed by one or more of the original referees, and acceptance of your manuscript will depend on your addressing satisfactorily the reviewers' major concerns. Please also note that Development will normally permit only one round of major revision. If it would be helpful, you are welcome to contact us to discuss your revision in greater detail. Please send us a point-by-point response indicating your plans for addressing the referees' comments, and we will look over this and provide further guidance.

Reviewer 1

Advance summary and potential significance to field

This paper provides a perspective on the epithelial-mesenchymal transition (EMT), a key biological process in development and cancer, by exploring how gene regulatory network (GRN) logic affects EMT transition dynamics. By leveraging a minimal three-node GRN model, the authors reveal that the choice of network logic—multiplicative (AND) versus additive (OR)—has profound implications for the stability and transitions between epithelial, mesenchymal, and hybrid E/M states. Their findings demonstrate that EMT dynamics are highly sensitive to how signals regulate transcription

© 2025. Published by The Company of Biologists under the terms of the Creative Commons Attribution License (https://creativecommons.org/licenses/by/4.0/).

factors, such as ZEB, through direct or indirect mechanisms, which can affect the accessibility and stability of hybrid states relevant to tissue remodeling and collective cell migration.

The paper makes a meaningful theoretical contribution to developmental biology by proposing that the choice of GRN logic is not merely a modeling detail but a fundamental determinant of cellular behavior during EMT. The results provide a conceptual framework that integrates mathematical modeling with biological processes, offering insights into how multistable landscapes emerge during development and how they might be perturbed in pathological contexts like cancer metastasis.

In particular, this study has potential relevance for developmental biologists as it highlights the importance of EMT hybrid states, which are implicated in morphogenesis, tissue plasticity, and embryonic cell migration. Furthermore, the work suggests experimental approaches for inferring network logic in live cells, paving the way for broader application to other developmental transitions driven by GRNs. My major issue of concern is that the study's technical focus on mathematical modeling limits its immediate accessibility to the majority of developmental biologists, its implications for understanding EMT regulation during development position it as a valuable contribution to the field. As a theoretical developmental biologist, I found challenging following the arguments in the paper.

Comments for the author

While the manuscript offers insights into the dynamics of EMT and GRN logic, its heavy technical focus and limited experimental integration. As it stands, it may be better suited to journals that specialize in systems biology, computational biology, or mathematical biology, such as: Bulletin of Mathematical Biology, Journal of Theoretical Biology, PLoS Computational Biology, or Mathematical Biosciences.

If the authors wish to strengthen the manuscript's fit for Development, they could:

- Expand the biological context and explicitly tie the findings to developmental processes beyond EMT (e.g., tissue morphogenesis, cell migration in embryogenesis).
- Emphasize general principles of GRN dynamics that could be applied to other developmental systems.
- Collaborate with experimentalists to provide supporting biological data.

Without these changes, the paper might indeed be perceived as too technical and narrow in scope for the journal's readership.

MAJOR POINTS FOR CONSIDERATION

(1) Clarify the Biological Context: While the mathematical and computational modeling is thorough, the biological implications could be more explicitly tied to broader developmental processes beyond EMT. For example, discussing how network logic might influence other developmental transitions, such as those involved in tissue morphogenesis or differentiation, would increase the paper's relevance to developmental biologists. They could also highlight more clearly how the insights into EMT dynamics can inform understanding of plasticity and cell state transitions during embryogenesis or tissue regeneration.

(2) Strengthen Experimental Connections: The proposed experimental designs are thoughtful, but they would benefit from more detailed descriptions of how they could be implemented in developmental systems beyond cancer cell lines (e.g., EMT during gastrulation or neural crest migration). They could also consider including a discussion of specific experimental models (e.g., organoids, embryonic systems, or developmental mutants) where these network logic predictions could be tested.

(3) Simplify and Focus the Technical Presentation: Some sections, particularly in the Results and Methods, are highly technical and dense. Simplifying the presentation of mathematical/technical details, while moving some of the deeper technical aspects to the Supplementary Information, could make the main text more accessible to a broad audience. I would also suggest to summarize

© 2025. Published by The Company of Biologists under the terms of the Creative Commons Attribution License (https://creativecommons.org/licenses/by/4.0/).

key results at the end of each subsection in the Results to help readers focus on the biological significance of the findings.

(4) Provide a More General Discussion of GRN Logic: The manuscript largely focuses on the role of network logic in EMT, but extending the discussion to consider how GRN logic might influence other multistable biological systems could make the findings more broadly relevant to the journal's readership. The authors could also emphasize how the framework developed in this study could serve as a foundation for analyzing other transitions in development, such as the switch between progenitor and differentiated cell states.

(5) Enhance Figure Annotations and Legends: The figures are well-designed, but some are challenging to interpret without referring extensively to the text. Adding annotations (e.g., arrows highlighting key bifurcation points or transitions) would improve their clarity.
They can also expand the figure legends to summarize the main biological takeaways from each figure.

(6) Address Experimental Validation: A key limitation of the study is the lack of direct experimental validation for the model predictions. While the authors suggest future experimental approaches, adding preliminary experimental data or simulations based on specific biological systems would strengthen the manuscript.

(7) Expand the Biological Discussion: The discussion is focused heavily on the computational findings and their implications for EMT. Expanding this to include more direct discussion of how these results might guide experimentalists studying EMT in a developmental context would improve the paper's impact. The authors could consider addressing whether the findings regarding hybrid E/M states and transition paths might have implications for processes such as collective cell migration during development.

(8) Explain Assumptions and Limitations: Provide a more explicit discussion of the assumptions underlying the GRN models and the potential limitations of focusing on AND/OR logic. Address how these assumptions might affect the generalizability of the findings to real biological systems.

(9) Clarify Relevance to Developmental Biology: While EMT is a critical process in both development and disease, the manuscript currently emphasizes cancer metastasis. Shifting the focus slightly to developmental contexts, such as neural crest migration, wound healing, or tissue plasticity during embryogenesis, would help align the manuscript more closely with the journal's scope.

(10) Expand the Technical Methodology: The explanation of the technical methodology could be expanded to better accommodate a developmental biology audience by providing basic definitions and concise digests of key mathematical concepts. The authors could also consider adding a figure that summarizes how their methodology was implemented step-by-step, which would make the approach more approachable for biologists. This manuscript bears resemblance to the Cell paper "Defining Network Topologies that Can Achieve Biochemical Adaptation" (Cell Volume 138, Issue 4, p760-773, August 21, 2009), and its accompanying preview (DOI: 10.1016/j.cell.2009.08.010). These papers succeeded in making complex mathematical approaches accessible to a biological readership. Adopting similar strategies, such as providing schematic overviews and simplified explanations, could enhance the accessibility of this manuscript.

Reviewer 2

Advance summary and potential significance to field

This manuscript explores the role of gene regulatory network (GRN) logic in epithelial-mesenchymal transition (EMT), a process fundamental to development and cancer metastasis. EMT is known to be controlled by a three-node GRN, which exhibits tristable behavior under certain conditions. The study investigates, through a mathematical model, how the choice of additive versus multiplicative regulation in this network influences steady states and transition dynamics.

© 2025. Published by The Company of Biologists under the terms of the Creative Commons Attribution License (https://creativecommons.org/licenses/by/4.0/).

The key result of the paper is that, while both logics can give rise to tristability, they differ in how they respond to perturbations. By analyzing these differences and comparing them with existing experimental data, the authors argue that the EMT network follows an additive logic. This finding is significant because, in the absence of direct experimental evidence, models are often constructed with arbitrary assumptions about network logic. The manuscript provides a systematic approach to distinguish between alternatives and suggests ways to infer network logic from experimental observations.

The work is methodologically rigorous and presents a thorough mathematical analysis of the EMT landscape. The results have implications not only for EMT but also for a broader class of biological systems where network motifs influence cell fate decisions. Given its clarity and relevance, the paper will be of interest to the readers of Development and makes a meaningful contribution to the field. I therefore recommend publication, provided that the authors address some concerns detailed below.

Comments for the author

One significant issue concerns the reproducibility of the stochastic simulations. The manuscript does not provide enough detail regarding the values of parameters such as V and sigma, which are critical in determining noise strength. Additionally, the notation \sigma_x(Y_t) in the supplement is somewhat misleading, as the main text describes the noise as additive. If the noise is indeed additive, then \sigma_x should be a constant rather than a function of the system's state. Furthermore, the role of the volumetric factor V should be clarified. If it effectively rescales variables, this would imply that different noise strengths apply to different species. It would be preferable to justify this explicitly rather than introducing an additional parameter whose effect remains unclear.

Another conceptual point that merits discussion is the nature of noise in the model. As Coomer et al. (2021) point from previous studies (e.g., Gillespie, 2000) have established, intrinsic noise is best captured by a multiplicative term. The authors should acknowledge that the linear noise term they introduce corresponds to extrinsic noise rather than intrinsic fluctuations. This is an acceptable modeling choice, particularly for testing robustness under mild noise conditions, but it should be made explicit to avoid confusion. The justification given that multiplicative noise might change the dynamics is not a valid consideration, on the contrary, it would be an argument to introduce more detailed noise models.

The presentation of some figures could also be improved. When printed, the gray shading for the monostable region in Figure 1 was not visible, which may indicate an issue with contrast. Additionally, the caption refers to 1U and 2U labeling for transitions, but these labels do not appear in the figure panels, making interpretation more difficult. Similarly, in Figures 4E and 4F, the differences between conditions appear small, raising the question of whether they are biologically meaningful. Given that parameter sets are chosen randomly, the perturbations applied are also random sets, meaning that comparing grouped perturbations may not provide significant additional insight. The interpretation of these panels should therefore be presented with more nuance (some systematic effects do appear to exist), or alternatively, they could be omitted if they do not provide essential information.

A structural change that would improve readability is the placement of Equations (1) and (2). These equations form the core of the mathematical framework and should be included in the main text rather than being relegated to the methods section. Readers who are comfortable with mathematical models will find them immediately informative, while others can skip over them without losing the overall message.

A brief discussion of hybrid logics, in which some regulatory inputs act additively while others act multiplicatively, would also be valuable. The study concludes that EMT follows an additive logic, but it would be useful to acknowledge that real biological networks often exhibit mixed logic. Recognizing this possibility would help clarify the broader applicability of the findings. Moreover, from the discussion presented, it would seem that the most relevant effect is wether negative regulations are additive or positive, and this can be discussed in a broader context than purely AND

© 2025. Published by The Company of Biologists under the terms of the Creative Commons Attribution License (https://creativecommons.org/licenses/by/4.0/).

or OR logics. To be clear, I am not asking for a whole new study of these cases, just a small discussion.

Finally, the manuscript would benefit from concrete examples of how additive and multiplicative regulatory logics emerge in biological systems. Sanford et al. (2020) provides useful discussions of both cases, linking additive regulation to independent recruitment of RNA polymerase and multiplicative regulation to thermodynamic models of transcription factor binding. While it is not necessary to discuss these models in detail, a brief reference to how such regulatory logics arise in different biological contexts would provide valuable context for readers who may not be familiar with these concepts.

In conclusion, this is an important and well-executed study that contributes meaningfully to our understanding of EMT and gene regulatory networks. Addressing the points above will further strengthen the manuscript, and I look forward to seeing it published in Development.

First revision

Author response to reviewers' comments

Reviewer 1

This paper provides a perspective on the epithelial-mesenchymal transition (EMT), a key biological process in development and cancer, by exploring how gene regulatory network (GRN) logic affects EMT transition dynamics. By leveraging a minimal three-node GRN model, the authors reveal that the choice of network logic—multiplicative (AND) versus additive (OR)—has profound implications for the stability and transitions between epithelial, mesenchymal, and hybrid E/M states. Their findings demonstrate that EMT dynamics are highly sensitive to how signals regulate transcription factors, such as ZEB, through direct or indirect mechanisms, which can affect the accessibility and stability of hybrid states relevant to tissue remodeling and collective cell migration.

The paper makes a meaningful theoretical contribution to developmental biology by proposing that the choice of GRN logic is not merely a modeling detail but a fundamental determinant of cellular behavior during EMT. The results provide a conceptual framework that integrates mathematical modeling with biological processes, offering insights into how multistable landscapes emerge during development and how they might be perturbed in pathological contexts like cancer metastasis.

In particular, this study has potential relevance for developmental biologists as it highlights the importance of EMT hybrid states, which are implicated in morphogenesis, tissue plasticity, and embryonic cell migration. Furthermore, the work suggests experimental approaches for inferring network logic in live cells, paving the way for broader application to other developmental transitions driven by GRNs. My major issue of concern is that the study's technical focus on mathematical modeling limits its immediate accessibility to the majority of developmental biologists, its implications for understanding EMT regulation during development position it as a valuable contribution to the field. As a theoretical developmental biologist, I found challenging following the arguments in the paper.

SUGGESTIONS TO AUTHORS
While the manuscript offers insights into the dynamics of EMT and GRN logic, its heavy technical focus and limited experimental integration. As it stands, it may be better suited to journals that specialize in systems biology, computational biology, or mathematical biology, such as: Bulletin of Mathematical Biology, Journal of Theoretical Biology, PLoS Computational Biology, or Mathematical Biosciences.

If the authors wish to strengthen the manuscript's fit for Development, they could:

© 2025. Published by The Company of Biologists under the terms of the Creative Commons Attribution License (https://creativecommons.org/licenses/by/4.0/).

- Expand the biological context and explicitly tie the findings to developmental processes beyond EMT (e.g., tissue morphogenesis, cell migration in embryogenesis).
- Emphasize general principles of GRN dynamics that could be applied to other developmental systems.
- Collaborate with experimentalists to provide supporting biological data.

Without these changes, the paper might indeed be perceived as too technical and narrow in scope for the journal's readership.

Response: We thank the reviewer for their detailed analysis and thoughtful comments. Following their suggestions, in the revision we have expanded on the biological context with several examples of models in development including a new section modeling inner cell mass differentiation, in doing so extending the theory to general principles of logic, and providing direct comparison between simulation results and data. We appreciate these suggestions as the paper is much stronger as a result. Point-by-point responses are given below.

1. Clarify the Biological Context: While the mathematical and computational modeling is thorough, the biological implications could be more explicitly tied to broader developmental processes beyond EMT. For example, discussing how network logic might influence other developmental transitions, such as those involved in tissue morphogenesis or differentiation, would increase the paper's relevance to developmental biologists. They could also highlight more clearly how the insights into EMT dynamics can inform understanding of plasticity and cell state transitions during embryogenesis or tissue regeneration.

Response: In the revision we have clarified the biological context by expanding upon the discussion and adding several new biological examples (with new analysis and simulations) to demonstrate the impact of logic on cell fate decisions in development.

Within EMT, we have clarified the relevance of our results to cases of EMT in development and in wound healing. Discussing differences in these systems with EMT as observed in cancer. Beyond EMT, we have added discussion of how logic impacts two models of cell fate decisions in development: one during neural crest development, and the other characterizing differentiation of cells of the inner cell mass (ICM) in the blastocyst. Here, cells differentiate into either epiblast or primitive endoderm under the control of a Gata6---Nanog feedback network, which previous work suggested is wired by a specific form of mixed logic. In the revision we recapitulated the previous results and went beyond this work by simulating ICM differentiation under two different types of mixed logic (more details below).

Through analysis of the resulting models via deterministic and stochastic simulations, we have shown that for more complex choices of mixed logic the central result of our work is upheld: that logic choice affects cell fates through the *dynamics*, even when the stability landscapes for different models appear the same or similar.

Finally, in the revision we have added further discussion about the generality of our results on regulatory network models and the decisive role that logic can play in the control of cell fates in these systems. We are grateful for the suggestion as extending the context beyond EMT has strengthened both our theoretical results regarding roles of network logic and the biological implications of the results on EMT and development systems.

© 2025. Published by The Company of Biologists under the terms of the Creative Commons Attribution License (https://creativecommons.org/licenses/by/4.0/).

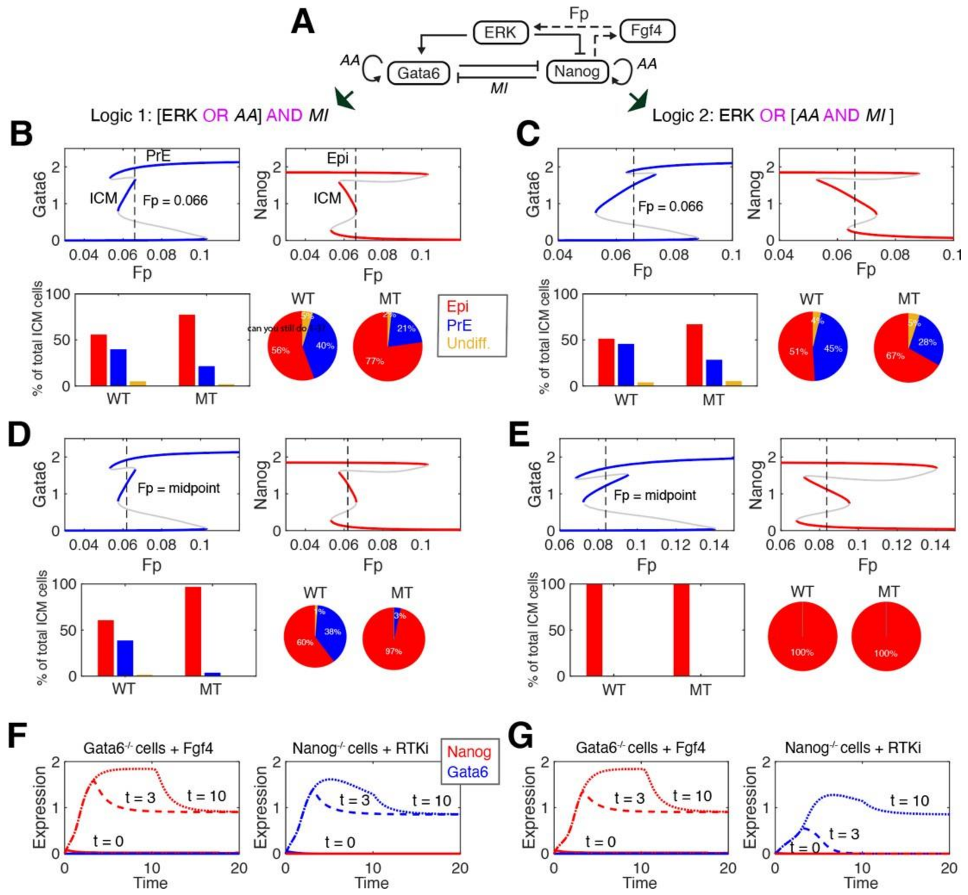

Fig. R1 (new Figure 6 in the revision) **Alternative mixed logic models can explain cell fate decisions in the ICM.**
A. Schematic of the model: intracellular mutual inhibition (MI) between Gata6 and Nanog, each experiencing auto activation (AA) and regulation from ERK. Dashed lines denote extracellular component: secretion of Fgf4 which activates ERK via the total extracellular Fgf4 (Fp). B. For Logic 1: tristable landscapes for Gata6 (blue; PrE fate) and Nanog (red, Epi fate) with respect to Fp for Logic 1. Lower panel: for stochastic simulations of 500 ICM cells, the proportion of cells committing to each fate are shown. Undifferentiated ICM cells (Undiff.) shown in yellow, and Fp = 0.066. C. For Logic 2: tristable landscapes for Gata6 (blue; PrE fate) and Nanog (red, Epi fate) with respect to Fp. Lower panel as for (A). Fp = 0.066. D. For Logic 1: landscapes for Gata6 and Nanog similar to (A) but with external Fgf4 levels at the midpoint of the ICM state (Fp = midpoint). E. As for panel (D) for Logic 2. F. The dynamics of ICM differentiation for Logic 1 in Gata6−/− cells with the addition of Fgf4 at different times [t ∈ (0, 3, 10)] (left); or in Nanog−/− cells with the addition of RTKi at different times [t ∈ (0, 3, 10)] (right). G. As for panel (D) for Logic 2.

2. Strengthen Experimental Connections: The proposed experimental designs are thoughtful, but they would benefit from more detailed descriptions of how they could be implemented in developmental systems beyond cancer cell lines (e.g., EMT during gastrulation or neural crest migration). They could also consider including a discussion of specific experimental models (e.g., organoids, embryonic systems, or developmental mutants) where these network logic predictions could be tested.

Response: We appreciate this suggestion, and in the revision we have expanded our discussion of experiments and experimental systems that can be used to test results on EMT network logic beyond cancer. Specifically, we have added new discussion on the role of logic in models of EMT controlled by regulatory networks in wound healing and in the neural crest. In this discussion, we also present experimental systems appropriate for testing the logic.

In the context of wound healing, a review of the literature revealed that GRHL2 plays a crucial

© 2025. Published by The Company of Biologists under the terms of the Creative Commons Attribution License (https://creativecommons.org/licenses/by/4.0/).

role as an EMT suppressor, is wired similarly to miR-200, and studies of cutaneous wound healing and scratch assays have shown that its over-expression is sufficient to initiate MET in the healing wound. We have added a discussion of how the results on logic extend to the context of EMT in wound healing, mediated by GRHL2, and how experimental scratch assays could be used to reveal the logical construction of the GRHL2---ZEB1 regulatory network.

In the context of EMT in the neural crest, we emphasize the importance of spatiotemporal regulation and the larger size of the EMT regulatory network. Thus, likely it will be necessary to quantitively measure gene/protein expression spatiotemporally (e.g. by live cell imaging) to study the impact of logical choices on models of EMT in this setting.
In the context of other cell fate decisions in the neural crest, an intriguing model in the literature proposes cyclical fate restriction as means to specify fates during neural crest differentiation. In this model, logic plays an important role in fate determination: whereby only with AND construction is a relatively even distribution of cell fates observed. However, further experimental evidence is needed here to confirm that alternative cell fate distributions are not observed during NCC differentiation. We propose that either in vivo (single-cell RNA sequencing) or in vitro (directed differentiation or gastruloid assays) could be used here to confirm the relative proportions of cell fates neural crest cells produce upon differentiation (new Results section: "*The role of logic in EMT during wound healing and ontogenesis*").

3. Simplify and Focus the Technical Presentation: Some sections, particularly in the Results and Methods, are highly technical and dense. Simplifying the presentation of mathematical/technical details, while moving some of the deeper technical aspects to the Supplementary Information, could make the main text more accessible to a broad audience. I would also suggest to summarize key results at the end of each subsection in the Results to help readers focus on the biological significance of the findings.

Response: We appreciate this request, and in the revision we have reduced the technical complexity across several sections. We have removed non-essential technical details, added simpler descriptions/clarifications where appropriate, and overall sought to simplify the results presentation, minimizing technical details wherever appropriate. We have moved one entire results section, "Analyzing properties of tristable EMT landscapes…" to the SI; as this was deemed more appropriate as supplementary details supporting the results.
We have also added summary paragraphs at the end of each remaining Results section.

4. Provide a More General Discussion of GRN Logic: The manuscript largely focuses on the role of network logic in EMT, but extending the discussion to consider how GRN logic might influence other multistable biological systems could make the findings more broadly relevant to the journal's readership. The authors could also emphasize how the framework developed in this study could serve as a foundation for analyzing other transitions in development, such as the switch between progenitor and differentiated cell states.

Response: In the revision, in clarifying and expanding upon the biological context (we have added several examples from development and regeneration), we have also considerably expanded upon the discussion of GRN network logic. Specifically:

1. We discuss how logical construction of the core miR-200---ZEB regulatory network is impacted as this network changes or grows in size (in wound healing or EMT in the neural crest);
2. We discuss the impact of logic on a model of neural crest differentiation that exhibits a Hopf bifurcation, i.e. oscillations can be created or destroyed, which shows how different choices of logic change the dynamics of the system (different cell fate biases via oscillations);
3. We performed new model analysis of a regulatory network for Gata6---Nanog: through analysis of alternative choices of mixed logic (combinations of AND & OR gates) we demonstrated the generalizability of our results on the EMT regulatory network.

We also agree that in general terms (i.e. in lieu of a specific regulatory network) our results on logic can inform cell state transitions in development. Cell differentiation from a progenitor to a differentiated cell state is often described mathematically by a model undergoing a saddle node bifurcation: the same type of bifurcation that we study extensively in light of logic in this

© 2025. Published by The Company of Biologists under the terms of the Creative Commons Attribution License (https://creativecommons.org/licenses/by/4.0/).

work. Thus, where models of cell differentiation exhibit similar bifurcations, our logical findings are likely relevant. We have added a discussion of such transitions in the Discussion of the revised manuscript.

5. Enhance Figure Annotations and Legends: The figures are well-designed, but some are challenging to interpret without referring extensively to the text. Adding annotations (e.g., arrows highlighting key bifurcation points or transitions) would improve their clarity. They can also expand the figure legends to summarize the main biological takeaways from each figure.

Response: We have updated the figures in light of these suggestions. Specifically

- In Fig. 1 the colors and shaded regions have been improved for clarity
- In Figs. 2 and 3 arrows added to show transitions as parameters perturbed by 25 or 50%
- In Fig. 4 we have improved the presentation of the hybrid state accessibility by performing a new analysis of saddle node points (updated Fig. 4E-F and accompanying text).
- In Fig. 5 the transition points have been highlighted with red boxes.

We have also sought to improve the figure captions such that these are self-contained and the figures can be read clearly without requiring additional explanation in the text.

6. Address Experimental Validation: A key limitation of the study is the lack of direct experimental validation for the model predictions. While the authors suggest future experimental approaches, adding preliminary experimental data or simulations based on specific biological systems would strengthen the manuscript.

Response: In the revision we have added a new section in which we analyze a model of inner cell mass differentiation, under the control of a Nanog---Gata6 regulatory network. Previous work has collected quantitative datasets on the dynamics of Nanog and Gata6 in blastocysts that allow us to directly compare the predictions of models of this regulatory network (specified by different logic) with the data (Bessonnard et al., Development, 2014). These models make specific predictions regarding the dynamics and the steady state distributions of cell fates under different assumptions of logic. Direct comparison of the simulation results and the data has shown us that: 1) multiple logical configurations are consistent with the data on wild type and homogeneous (Nanog or Gata6 mutant dynamics; 2) only one logic is consistent with the proportions of cell fates observed if we assume that the committed cell fates (epiblast or primitive endoderm) must be accessible from all ICM states (new Results section pg. 14 and new Fig. 6).

More generally, through several lines of evidence in the revision we have emphasized that the key conclusion of our work is that caution must be taken in the construction of models from regulatory networks given a choice of logic. Hence the focus on experimental design: experimental validation can be performed only in light of a certain hypothesis. Our results give development biologists means with which to compare and distinguish regulatory network logics in models *given* a certain hypothesis.

7. Expand the Biological Discussion: The discussion is focused heavily on the computational findings and their implications for EMT. Expanding this to include more direct discussion of how these results might guide experimentalists studying EMT in a developmental context would improve the paper's impact. The authors could consider addressing whether the findings regarding hybrid E/M states and transition paths might have implications for processes such as collective cell migration during development.

Response: In the revision we have expanded upon the biological discussion both in terms of EMT and other developmental systems via two new Results sections. We have concordantly reduced the technical/computational discussion in the main text considerably. We now include more discussion of how our results can guide experimentalists working on EMT in development or regeneration in the revision through a new Results section. Specific additions in the new Results sections include:

- A discussion of the role of GRHL2 in controlling EMT in wound healing, and how its

© 2025. Published by The Company of Biologists under the terms of the Creative Commons Attribution License (https://creativecommons.org/licenses/by/4.0/).

inhibition of ZEB1 in the regulatory network is likely wired by AND logic in light of experimental data.
- A discussion of EMT in the neural crest, and how in this context larger regulatory network make mixed logical configurations more likely.
- New computational modeling, analysis, and discussion of the role of logic in a regulatory network model of early blastocyst cell fate decisions (in the differentiating ICM). Further details in answers to Q1 and Q6 above and in the Results section: "*The effects of logic control the dynamics of cell fate specification during differentiation of the inner cell mass.*"

8. Explain Assumptions and Limitations: Provide a more explicit discussion of the assumptions underlying the GRN models and the potential limitations of focusing on AND/OR logic. Address how these assumptions might affect the generalizability of the findings to real biological systems.

Response: In the revision we have added further explanation of the modeling assumptions in the Methods and limitations of the model in the Discussion. In particular, we have sought to highlight:

1) the specific assumptions of the EMT model that must be taken into account (e.g. that transcription is not considered in the model); 2) the more general assumptions that must be taken into account in the choice of logic when constructing models of regulatory networks. For the latter, we discuss the role that pure vs. mixed logic plays, how the likelihood of mixed logic will increase with the size of the network, and how larger networks than the simple 'motifs' considered here can generate higher order stability (i.e. four or more stable steady states) with many more SN points and subsequently many more transition paths across the landscape.

9. Clarify Relevance to Developmental Biology: While EMT is a critical process in both development and disease, the manuscript currently emphasizes cancer metastasis. Shifting the focus slightly to developmental contexts, such as neural crest migration, wound healing, or tissue plasticity during embryogenesis, would help align the manuscript more closely with the journal's scope.

Response: We agree and in the revision we have shifted the focus. For full details please see answers to the questions above. To summarize: we have expanded the scope of the models considered to include discussion and analysis of models in the context of EMT in development and regeneration and to non-EMT cell fate decisions during development. We have discussed EMT in the context of neural crest fate decisions and wound healing; we have also cell fate decisions in the neural crest differentiation via oscillatory models of cell fate decisions. We have added substantive new analysis of a tristable network that controls the differentiation of the inner cell mass in the blastocyst, which corroborates and extends our results on the impact of logic in the EMT network: in the inner cell mass, alternative models of mixed logical construction are consistent with data that describe the dynamics of Gata6 and Nanog expression in the blastocyst under wild type and various mutant conditions.

10. Expand the Technical Methodology: The explanation of the technical methodology could be expanded to better accommodate a developmental biology audience by providing basic definitions and concise digests of key mathematical concepts. The authors could also consider adding a figure that summarizes how their methodology was implemented step-by-step, which would make the approach more approachable for biologists. This manuscript bears resemblance to the Cell paper" Defining Network Topologies that Can Achieve Biochemical Adaptation" (Cell Volume 138, Issue 4, p760-773, August 21,2009), and its accompanying preview (DOI: 10.1016/j.cell.2009.08.010). These papers succeeded in making complex mathematical approaches accessible to a biological readership. Adopting similar strategies, such as providing schematic overviews and simplified explanations, could enhance the accessibility of this manuscript.

Response: In the revision, we have followed the reviewer's suggestion. We agree that such an exposition can assist with the presentation of the technical methodology to a broader audience consisting of both theoretical and experimental biologists. To orient the reader towards our analysis of accessible cell states in multistable models and how these are impacted by logic, we have added a new figure (Fig. 1A) that details step-by-step how one goes about constructing an ODE model from a gene network where a target ("B") receives multiple inputs: we show the different forms of equations for AND vs OR logic, and show how different logics can produce the

© 2025. Published by The Company of Biologists under the terms of the Creative Commons Attribution License (https://creativecommons.org/licenses/by/4.0/).

same unperturbed bifurcation landscape (blue curve) and yet produce very different bifurcation landscapes – and thus cell fate distributions – when perturbed (red curve).
This figure helps to highlight the crucial point of our study: that comparison of perturbations & dynamics of models constructed with different logic is rare and yet can be crucial, since the choice of logic can determine outcomes (i.e. cell fates) in surprising and unpredictable ways.

Reviewer 2
This manuscript explores the role of gene regulatory network (GRN) logic in epithelial-mesenchymal transition (EMT), a process fundamental to development and cancer metastasis. EMT is known to be controlled by a three-node GRN, which exhibits tristable behavior under certain conditions. The study investigates, through a mathematical model, how the choice of additive versus multiplicative regulation in this network influences steady states and transition dynamics.

The key result of the paper is that, while both logics can give rise to tristability, they differ in how they respond to perturbations. By analyzing these differences and comparing them with existing experimental data, the authors argue that the EMT network follows an additive logic. This finding is significant because, in the absence of direct experimental evidence, models are often constructed with arbitrary assumptions about network logic. The manuscript provides a systematic approach to distinguish between alternatives and suggests ways to infer network logic from experimental observations.

The work is methodologically rigorous and presents a thorough mathematical analysis of the EMT landscape. The results have implications not only for EMT but also for a broader class of biological systems where network motifs influence cell fate decisions. Given its clarity and relevance, the paper will be of interest to the readers of Development and makes a meaningful contribution to the field. I therefore recommend publication, provided that the authors address some concerns detailed below.

Response: We thank the reviewer for their thorough evaluation of this manuscript, and we appreciate their enthusiasm for its findings. Below we provide a point-by-point response to the specific questions raised.

1. One significant issue concerns the reproducibility of the stochastic simulations. The manuscript does not provide enough detail regarding the values of parameters such as V and sigma, which are critical in determining noise strength. Additionally, the notation \sigma_x(Y_t) in the supplement is somewhat misleading, as the main text describes the noise as additive. If the noise is indeed additive, then \sigma_x should be a constant rather than a function of the system's state. Furthermore, the role of the volumetric factor V should be clarified. If it effectively rescales variables, this would imply that different noise strengths apply to different species. It would be preferable to justify this explicitly rather than introducing an additional parameter whose effect remains unclear

Response: We appreciate that in the first submission the notation was slightly confusing. In the revision we have ensured that everything is explained and justified sufficiently well. NB: in the revision we have updated the main figure to multiplicative noise (see Q2 below), thus we no longer use the volumetric scaling factor in the main text. The additive noise simulation is still included for comparison as an SI figure.
To clarify the other updated points

- To avoid any confusion, we have replaced the notation used previously (!!(#") to denote the general noise model of the SDE) with %(#"), i.e. %(#") = ! for additive noise and %(#") = !#" for multiplicative noise, with constant noise parameter !.
- In EMT modeling, we used a value for the noise strength of ! = 0.01 for multiplicative noise and ! = 1 for additive noise.
- In the ICM differentiation model, we used a value of ! = 0.1 for the multiplicative noise.

2. Another conceptual point that merits discussion is the nature of noise in the model. As Coomer et al. (2021) point from previous studies (e.g., Gillespie, 2000) have established, intrinsic noise is best captured by a multiplicative term. The authors should acknowledge that the linear noise term they introduce corresponds to extrinsic noise rather than intrinsic

© 2025. Published by The Company of Biologists under the terms of the Creative Commons Attribution License (https://creativecommons.org/licenses/by/4.0/).

fluctuations. This is an acceptable modeling choice, particularly for testing robustness under mild noise conditions, but it should be made explicit to avoid confusion. The justification given that multiplicative noise might change the dynamics is not a valid consideration, on the contrary, it would be an argument to introduce more detailed noise models.

Response: We thank the reviewer for raising this point, which we agree is important, and also rather nuanced. We agree that exploring the stochastic dynamics of EMT network models with both additive and multiplicative noise models in the SDE is worthwhile. In the revision, we have performed this analysis.

We found that for both noise models, our ability to distinguish between EMT network logics by experimental design is upheld. That is, for relatively low additive or multiplicative noise, the inequalities presented in Fig. 5 are upheld, and the logical wiring of the EMT network can be inferred in live cells using increasing doses of TGF-ß and perturbed network edges (see new Fig. 5 for multiplicative noise and Fig. S9 A-D for additive noise).

Notably, if the magnitude of the noise under the multiplicative SDE model is increased substantially, we observed changes in the locations of the steady states inferred by taking the mean of the trajectories, in line the distortion of the landscape reported in Coomer et al. (2021). At such noise levels, random transitions between all three stable states become likely and thus distinct attractor states are lost. We present this analysis of the role of high noise of the bifurcation landscapes for additive noise (small effects to stable states) or multiplicative noise (large effects) in a new SI figure (Fig. S9E).

3. The presentation of some figures could also be improved. When printed, the gray shading for the monostable region in Figure 1was not visible, which may indicate an issue with contrast. Additionally, the caption refers to 1U and 2U labeling for transitions, but these labels do not appear in the figure panels, making interpretation more difficult. Similarly, in Figures 4E and 4F, the differences between conditions appear small, raising the question of whether they are biologically meaningful. Given that parameter sets are chosen randomly, the perturbations applied are also random sets, meaning that comparing grouped perturbations may not provide significant additional insight. The interpretation of these panels should therefore be presented with more nuance (some systematic effects do appear to exist), or alternatively, they could be omitted if they do not provide essential information.

Response: In the revision, we have replaced the gray shading in Fig. 1 with more visible colors. We have also added the transition types in the figure panel titles. Fig 1 updates to these panels are shown below:

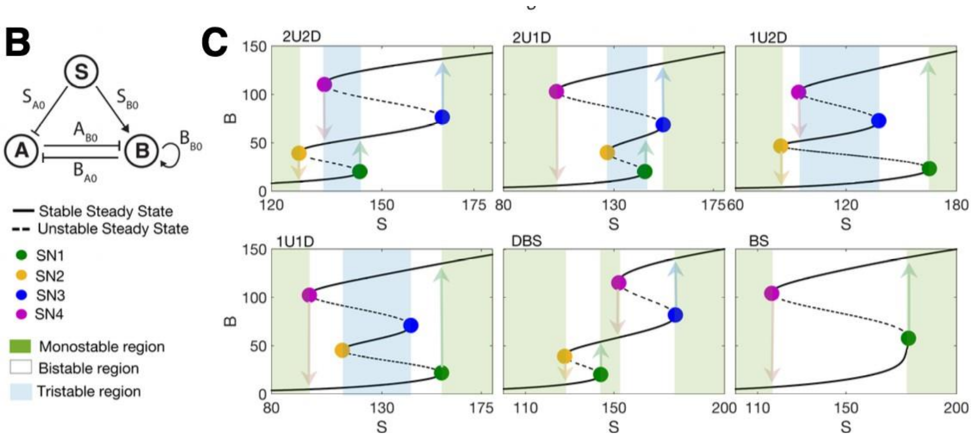

Regarding Fig. 4E-F, we agree with the reviewer that grouping models by tristable type was not the clearest way to present accessibility to the hybrid state since the accessibility of the state can change even when its type does not. In the revision, we have replaced this with a new analysis of the accessibility of the hybrid states (new Fig. 4E-F). We calculated the difference (SN3 - SN1) for each model after direct or indirect perturbations to assess changes in the accessibility of the hybrid state during *EMT*, and likewise the difference (SN4 - SN2) to quantify

© 2025. Published by The Company of Biologists under the terms of the Creative Commons Attribution License (https://creativecommons.org/licenses/by/4.0/).

changes in the accessibility of the hybrid state during the reverse MET. In both cases, larger positive values indicate a more accessible hybrid state.
The results show that during MET increasing the indirect regulation increases accessibility to the hybrid state in the AND models and decreases accessibility in the OR models. This means, that when miR-200 is overexpressed, the mesenchymal cells will quickly access the hybrid state and undergo MET in the AND models but *not* for the OR models: here they stay in mesenchymal states for longer (and have larger M states; see Fig 4D). This leads us to suggest that AND models are more consistent with experimental observations, where cells undergo MET with miR-200 overexpression in mesenchymal populations.

4. A structural change that would improve readability is the placement of Equations (1) and (2). These equations form the core of the mathematical framework and should be included in the main text rather than being relegated to the methods section. Readers who are comfortable with mathematical models will find them immediately informative, while others can skip over them without losing the overall message.

Response: We agree with the reviewer's suggestion, and we have added the equations of both AND and OR logic models at the start of the Results.

5. A brief discussion of hybrid logics, in which some regulatory inputs act additively while others act multiplicatively, would also be valuable. The study concludes that EMT follows an additive logic, but it would be useful to acknowledge that real biological networks often exhibit mixed logic. Recognizing this possibility would help clarify the broader applicability of the findings. Moreover, from the discussion presented, it would seem that the most relevant effect is whether negative regulations are additive or positive, and this can be discussed in a broader context than purely AND or OR logics. To be clear, I am not asking for a whole new study of these cases, just a small discussion.

Response: This is an important point, we agree that – while the EMT networks analyzed are comprised with exclusively AND or OR logic – mixed logic is also possible and as GRNs grow larger, hybrid logics will become likely.

In revising the manuscript, we have added discussion and analysis of several new GRNs in the context of EMT beyond cancer (wound healing or neural crest) and non-EMT cell fate decisions in embryogenesis. In several of these examples networks are constructed with mixed logic, including in the inner cell mass differentiation network, where we compare the effects of alternative assumptions of mixed logic and present the results in a new main figure (Fig. 6). To summarize briefly the results: as for the EMT networks, we found that different logics can lead to indistinguishable bifurcation landscapes for unperturbed models, but that when models are then perturbed in various ways, different landscapes will result, highlighting the importance of choosing logic carefully.

Finally, to clarify in the EMT network, both negative and positive regulations are impacted by logic: in the figure below the negative regulations in red (S -| A and A -| B) are sensitive to logic, but so is the positive regulation from S to B (S □ B). On the other hand, as we showed in the results, the negative regulation B -| A is *insensitive* to the choice of logic.

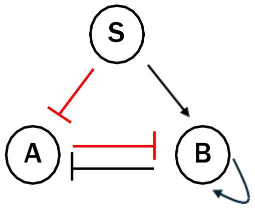

6. Finally, the manuscript would benefit from concrete examples of how additive and multiplicative regulatory logics emerge in biological systems. Sanford et al. (2020) provides useful discussions of both cases, linking additive regulation to independent recruitment of RNA polymerase and multiplicative regulation to thermodynamic models of transcription factor binding. While it is not necessary to discuss these models in detail, a brief reference to how

© 2025. Published by The Company of Biologists under the terms of the Creative Commons Attribution License (https://creativecommons.org/licenses/by/4.0/).

such regulatory logics arise in different biological contexts would provide valuable context for readers who may not be familiar with these concepts.

In conclusion, this is an important and well-executed study that contributes meaningfully to our understanding of EMT and gene regulatory networks. Addressing the points above will further strengthen the manuscript, and I look forward to seeing it published in Development.
Response: In the revision we have added a discussion of the motivation behind additive or multiplicative models. The paragraph below has been added to the start of the Results (third paragraph of the first Results Section):

In constructing an ODE model for this network, one must choose whether gene regulations combine additively or multiplicatively (Fig. 1A). This choice can be approached either phenomenologically or mechanistically, where in the former case one can use experimental observations of phenotypes to infer logic (as is done e.g. in (Bessonnard et al, 2014); in the latter case constructing models from first principles via mechanisms of transcription: models that appeal to thermodynamic principles to describe the action of RNA polymerase lead to multiplicative regulation (Bintu et al, 2005); models that alternatively assume transcription factors more commonly bind independently to regulate transcription lead to additive regulation (Sanford et al, 2020).

Second decision letter

MS ID#: dev.204583R1

MS TITLE: Transition paths across the EMT landscape are dictated by network logic

AUTHORS: Anupam Dey and Adam L. MacLean

Dear Adam,

I am happy to tell you that your manuscript has been accepted for publication in Development, pending our standard publication integrity checks.

Reviewer 2

The authors have done a huge and successful effort to improve the manuscript following the Reviewer's feedback. They have included whole new case studies to better connect the abstract theory to specific cases and exemplify its utility and validity. Technical correction, clarity of presentation and deepness of discussion are all improved. I recommend acceptance.

© 2025. Published by The Company of Biologists under the terms of the Creative Commons Attribution License (https://creativecommons.org/licenses/by/4.0/).
